# Lithium‐Charged Gold Nanoparticles: A New Powerful Tool for Lithium Delivery and Modulation of Glycogen Synthase Kinase 3 Activity

**DOI:** 10.1002/adma.202513858

**Published:** 2025-09-29

**Authors:** Antonio Buonerba, Giulia Puliatti, Domenica Donatella Li Puma, Bruno Bandiera, Beatrice Cannata, Maria Elena Marcocci, Nicolina Castagno, Irene Contento, Salvatore Impemba, Mariarosa Scognamiglio, Rocco Di Girolamo, Vincenzo Naddeo, Patrizia Canton, Carmine Capacchione, Laura Sposito, Martina Albini, Francesco Pastore, Silvia Baroni, Alfonso Grassi, Claudio Grassi, Roberto Piacentini

**Affiliations:** ^1^ Department of Chemistry and Biology *“Adolfo Zambelli”* University of Salerno Via Giovanni Paolo II Fisciano SA 84084 Italy; ^2^ Department of Neuroscience Università Cattolica del Sacro Cuore Largo F. Vito 1 Rome 00168 Italy; ^3^ Fondazione Policlinico Universitario A. Gemelli IRCCS Largo A. Gemelli 8 Rome 00168 Italy; ^4^ Department of Public Health and Infectious Diseases Sapienza University of Rome Piazzale Aldo Moro 5 Rome 00185 Italy; ^5^ Department of Industrial Engineering University of Salerno Via Giovanni Paolo II Fisciano SA 84084 Italy; ^6^ Department of Chemical Sciences University of Naples Federico II Via Cintia, 21 Naples 80126 Italy; ^7^ Department of Civil Engineering University of Salerno Via Giovanni Paolo II Fisciano SA 84084 Italy; ^8^ Department of Molecular Sciences and Nanosystems University Ca’ Foscari Venezia Dorsoduro 3246 Venezia 30123 Italy; ^9^ Department of Basic biotechnological sciences intensivological and perioperative clinics Università Cattolica del Sacro Cuore Largo F. Vito 1 Rome 00168 Italy

**Keywords:** Alzheimer's disease, bipolar disorder, glycogen synthase kinase 3, gold nanoparticle, HSV‐1 infection, lithium

## Abstract

***“A Trojan Horse for Lithium Delivery”***. Lithium has important pharmacological applications, although its use is severely limited due to its narrow therapeutic window of administrable concentrations. This study presents a novel nanocarrier for this metal cation based on glutathione‐stabilized gold nanoparticles (LiG‐AuNPs), which enable targeted release of lithium. LiG‐AuNPs are easily synthesized, with dimensions of ≈2 nm, with a lithium loading of 2 wt%. These particles show a tendency to aggregate and a narrow size distribution. Aggregates of LiG‐AuNPs (a‐LiG‐AuNPs) are non‐toxic to cells at concentrations lower than 2 mg mL^−1^ and are rapidly internalized into cells, where they release lithium in the cytosol through cation exchange, effectively modulating Glycogen Synthase Kinase‐3 (GSK‐3β) activity, especially the β isoform. Administration of a‐LiG‐AuNPs in murine models increased the inhibitory phosphorylation of GSK‐3β at Ser9. Intranasal administration of a‐LiG‐AuNPs modulated GSK‐3β activity in the brain, particularly in the hippocampus, without significantly altering plasma lithium levels, even when the administration lasted several months. LiG‐AuNPs thus represent a powerful tool for the targeted administration of lithium, enhancing its therapeutic effects in modulating GSK‐3β. They have potential applications for treating illnesses depending on GSK‐3β (hyper)activation, such as mood disorders, Alzheimer's disease, and viral infections.

## Graphical abstract

This study introduces glutathione‐stabilized gold nanoparticles for targeted lithium delivery (LiG‐AuNPs), enabling the controlled modulation of Glycogen Synthase Kinase‐3 (GSK‐3β). These non‐toxic, 2‐nm particles release lithium intracellularly, effectively inhibiting GSK‐3β in the brain without significant plasma lithium alterations. LiG‐AuNPs show potential for treating GSK‐3β‐related conditions like mood disorders, Alzheimer's disease, and viral infections through prolonged, targeted administration.

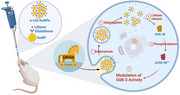

## 1 Introduction

Gold nanoparticles (AuNPs) have attracted considerable interest from both academic and industrial communities in designing tailored nanodevices for personalized medicine due to the unique chemical and physical properties of nanostructured gold. As a matter of fact, AuNPs are biologically non‐toxic and have been used as a scaffold for delivering drugs and therapeutics anchored to their surface via covalent or electrostatic interactions.^[^ ^1^ ^]^ Moreover, these systems are responsive to external physical and chemical stimuli, enabling the setup of nanodevices for innovative applications.^[^ ^2^ ^]^ However, despite the great potential, the selective and effective targeting of functional AuNPs still requires remarkable research efforts.

Recently, we developed glutathione (GSH)‐coated AuNPs (G‐AuNPs) with a narrow diameter size distribution of ≈2 nm that, in aqueous media, spontaneously produce aggregates of ≈100–300 nm.^[^ ^3^ ^]^ These NP aggregates demonstrated the ability to rapidly internalize in mammalian cells, as shown in human hepatocarcinoma HepG2 cells, human neuroblastoma SH‐SY5Y cells, and murine astrocytes. The adopted synthetic procedure employs alkali metal hydroxides (NaOH or LiOH) and the corresponding chloride salts for precipitation in hydroalcoholic media, affording the formation of metal alkali‐functionalized G‐AuNPs, where the metal cation acts as a reversible and highly fluxional cross‐linking point between the AuNPs. On the contrary, the singularly dispersed AuNPs remain anchored to the cell membrane, acting as fluorescent probes.^[^ ^4^ ^]^ Recently, we have proven that the aggregates of G‐AuNPs (*a*‐G‐AuNPs) functionalized with the dansyl chromophore (*a*‐DG‐AuNPs) and stabilized by the electrostatic interaction with sodium cations are highly thermally responsive under excitation with a pulsed laser emitting in the near‐infrared spectral region (NIR; λ = 800 nm). The efficient penetration of this radiation throughout the tissues without damaging healthy cells makes this nanodevice a powerful tool for the photoablation of cancer cells.^[^ ^3^, ^5^ ^]^ Additionally, *a*‐G‐AuNPs can be used as efficient carriers of metal cations in cells. Among them, lithium ions are particularly interesting from a biological perspective. Indeed, the lithium cation (Li^+^) is very effective in modulating/inhibiting the activity of the hub enzyme, glycogen synthase kinase 3 (GSK‐3). GSK‐3 is a Ser/Thr kinase that exists in two similar isoforms, α and β, involved in various intracellular signaling pathways that regulate several cellular processes, including, but not limited to, cell proliferation and differentiation, apoptosis, and immune response.^[^ ^6^ ^]^ From a pathological perspective, GSK‐3 activation, particularly its β isoform (GSK‐3β), has been linked to several diseases.^[^ ^7^ ^]^ GSK‐3 plays a crucial role in the etiology of mood disorders, for which lithium represents one of the elective therapeutic agents stabilizing mood, for example, in Bipolar Disorders (BD).^[^ ^8^ ^]^ In this view, GSK‐3 has been identified as a putative neuropsychiatric biomarker, allowing for differential diagnosis between Major Depressive Disorder (MDD) and BD.^[^ ^8^ ^]^ This kinase is also involved in several neurological/neurodegenerative diseases, such as Alzheimer's disease (AD) and other tauopathies, as well as Parkinson's disease (PD).^[^ ^9^ ^]^ In fact, it is the primary kinase determining tau (hyper)phosphorylation (p‐Tau) at several amino acids (e.g., Ser 199, Thr 205, Ser 396),^[^ ^10^ ^]^ responsible for tangle formation. It is also involved in the phosphorylation of the amyloid precursor protein (APP) at Thr668, leading to the proteolytic cleavage that results in the production of amyloid‐β peptide (Aβ).^[^ ^11^, ^12^ ^]^ Notably, p‐Tau (forming neurofibrillary tangles) and Aβ (forming amyloid plaques) are the two main molecular hallmarks of AD. GSK‐3β also regulates α‐synuclein pathology and is active in the striatum of patients with PD.^[^ ^9^ ^]^ Finally, several studies also demonstrated the crucial role of GSK‐3 in the phosphorylation and/or activation of proteins and receptors involved in the attachment, entry, and replication of several DNA and RNA viruses into cells, such as Herpes simplex virus type 1 (HSV‐1), that has been associated to the occurrence of sporadic AD when it reaches the brain,^[^ ^13^, ^14^, ^15^ ^]^ as well as various coronaviruses, including the SARS‐CoV‐1 and the SARS‐CoV‐2 responsible for the recent COVID‐19 pandemics.^[^ ^16^, ^17^, ^18^, ^19^ ^]^

Given the importance of GSK‐3β, especially in neuropsychiatric, neurodegenerative, and infectious diseases, numerous efforts have been made to identify molecules that can modulate it. Notably, GSK‐3 inhibition mainly depends on Ser phosphorylation in positions 21 and 9 for isoforms α and β, respectively. However, some stimuli may further activate this kinase by phosphorylation at Tyr279/216 (for GSK‐3α/β, respectively).^[^ ^11^, ^20^ ^]^ To date, several GSK‐3 inhibitors have been developed that may be categorized into four classes: *i*) metal cations, *ii*) ATP competitive, *iii*) allosteric non‐ATP competitive, and *iv*) substrate competitive.^[^ ^7^ ^]^ Although most newly developed/discovered GSK‐3 inhibitors belong to the second family, Li^+^ can still be considered one of the most powerful and clinically used GSK‐3 inhibitors. Indeed, very few GSK‐3‐inhibiting compounds, other than lithium salts (i.e., lithium carbonate), reached the stage of clinical trials, mainly because of the difficulty of reaching specifically the target organs (e.g., the brain). On the contrary, lithium salts have long been used in clinical practice for treating neuropsychiatric disorders. Indeed, the first reports date back to 1870,^[^ ^21^ ^]^ but the therapeutic use of these drugs was strongly limited by the toxicity of this metal cation.^[^ ^22^, ^23^, ^24^, ^25^ ^]^ Actually, lithium chloride, which is typically used for studies of in vitro inhibition of GSK‐3 at concentrations in the range of 5–20 mEq L^−1^, is highly toxic (up to be lethal) for humans at such doses.^[^ ^21^, ^26^ ^]^ To date, lithium carbonate is an elective, FDA‐approved, commercial drug administrable as oral tablets for the treatment of mood disorders and BD in particular; however, several unwanted side effects, mainly affecting the kidneys and thyroid, limit its wide therapeutic application, especially in older people.^[^ ^27^ ^]^ The range of lithium concentration in plasma, assuring an acceptable risk/benefit ratio in patients with mood disorders, is indeed 0.8–1.2 mEq L^−1^. Besides mood disorders, recent studies also suggested the efficacy of lithium carbonate against cognitive decline in mild cognitive impairment patients^[^ ^28^ ^]^ and COVID‐19 severity.^[^ ^29^ ^]^ However, the therapeutic doses of lithium required in these cases may need to be higher than those typically allowed based on the risk/benefit ratio. Therefore, identifying novel lithium administration routes that reach the target organ(s) (e.g., the brain) with high specificity and without causing toxicity in other organs would be highly desirable.

Here, we report the development and the potential applications of a highly versatile, safe, and effective tool for inhibiting the activity of GSK‐3β via intracellular release of Li^+^ driven by spherical gold nanoparticles functionalized with glutathione and charged with lithium cations at the outer corona (LiG‐AuNPs). This nanodevice allows: *i*) reducing the extracellular Li^+^ concentration efficacious for GSK‐3 inhibition; *ii*) assuring the delivery of lithium into the brain if administered intranasally, thus likely inhibiting GSK‐3 in situ without passing through systemic administration, i.e., blood circulation. Both these features should likely avoid the unwanted side effects due to the overdosage of lithium. Given the important potential applications of the developed nanocarrier, the authors patented the LiG‐AuNPs.^[^ ^30^ ^]^

## 2 Results

### 2.1 Synthesis and characterization of a‐LiG‐AuNPs

**Figure 1:**
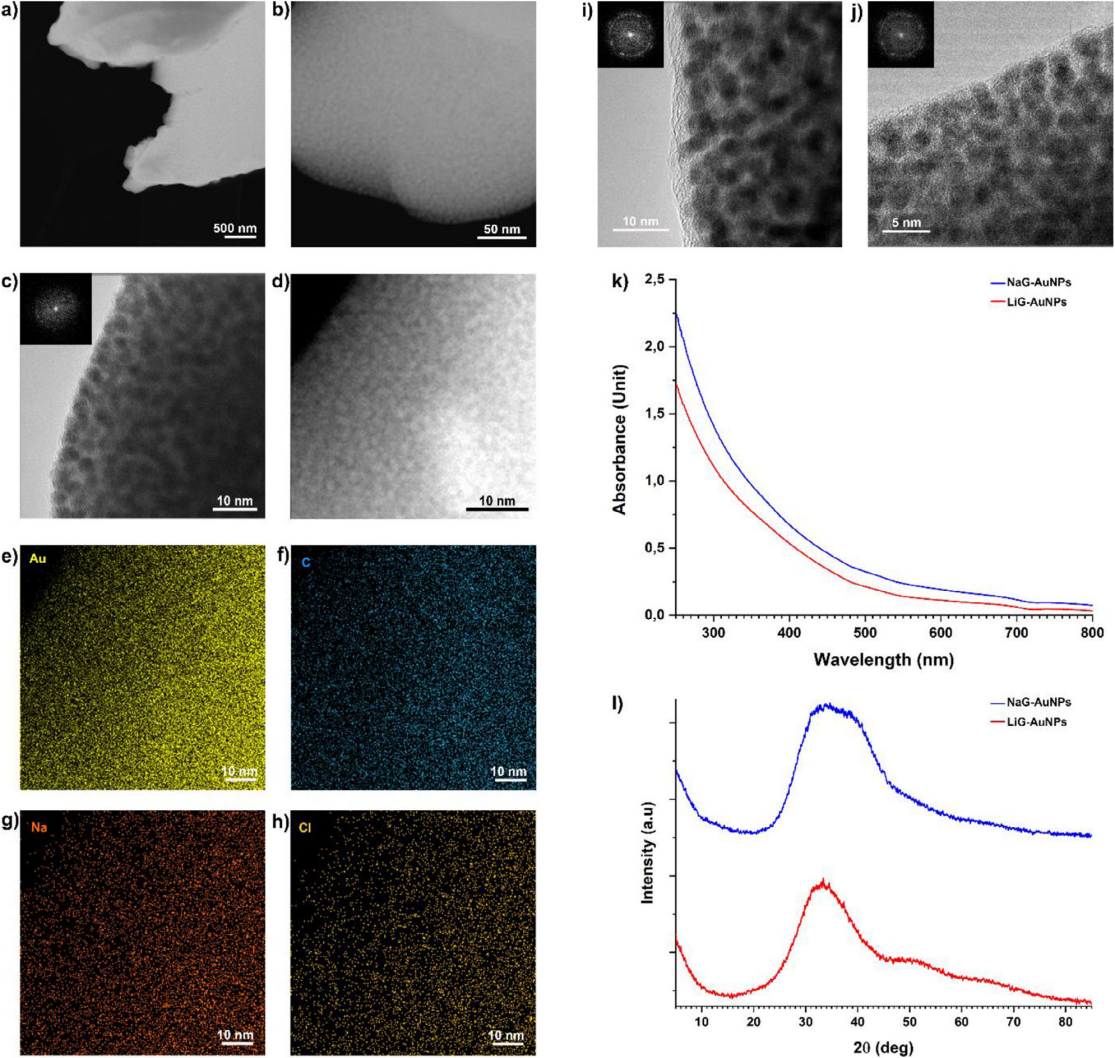
Structural characterization of LiG‐AuNPs and NaG‐AuNPs. a,b) STEM HAADF and c,d) HRTEM images with the corresponding Hanning Masked FFT pattern (c) of a‐NaG‐AuNPs. e–h) Gold, carbon, sodium, and chloride distributions by TEM‐EDS (from the area in micrograph d). i,j) HR‐TEM images of LiG‐AuNPs with the corresponding Hanning Masked FFT patterns. k) UV–Vis spectra and l) WAXD patterns for NaG‐AuNPs and LiG‐AuNPs.

Lithium‐functionalized spherical gold nanoparticles (LiG‐AuNPs) were synthesized starting from a procedure similar to that previously used for the sodium‐charged analogs (NaG‐AuNPs).^[^ ^3^ ^]^ Briefly, tetrachloroauric acid, reduced glutathione (GSH), and lithium hydroxide were dissolved in sequence in a hydroalcoholic solution, followed by the addition of sodium borohydride, which caused the reduction of the gold precursor to metallic nanoparticles. LiG‐AuNPs were recovered from the colloidal suspension by dilution with water/methanol solvent mixture and salt‐induced precipitation with lithium chloride within c.a. 2 days in an Imhoff cone that facilitates the recovery of pure aggregates. This procedure allows easy recovery of the AuNPs with monomodal size distribution and simple purification from the by‐products and lithium chloride excess. The transmission electron microscopy and scanning transmission electron microscopy (TEM/STEM) analyses of the LiG‐AuNPs showed a very narrow size distribution of spherical and crystalline AuNPs with an average diameter of 2 nm (**Figure** **1**a–d). The formation of small nanoparticles in the whole NaG‐AuNPs and LiG‐AuNPs samples was confirmed by UV‐visible spectroscopy (UV–Vis) and wide‐angle x‐ray scattering (WAXD) analysis. Both samples showed the absence of surface plasmon resonance (SPR) at a wavelength of ≈520 nm in the UV–Vis spectra (Figure 1k) and very broad x‐ray reflections in the WAXD patterns (Figure 1l) at 2θ angles of 35–40 °, indicative of AuNPs with sizes smaller than 5 nm.^[^ ^3^, ^31^, ^32^ ^]^ The formation of nanoparticle aggregates stabilized by electrostatic interaction with the metal cation at the surface was assessed by electron microscopy (TEM‐EDS) analysis (Figure 1e–h). Gold, carbon, sodium, and the corresponding counter‐anion chloride were found uniformly dispersed in the AuNPs aggregate in the solid state (Figure 1e–h). LiG‐AuNPs also showed a very small NP size of ≈2 nm with a very narrow size distribution (Figure 1i,j). The identification of the lithium(I) by STEM‐EDS was not possible for LiG‐AuNPs because of the low intensity of the x‐ray signal for this metal with a low atomic number.^[^ ^33^ ^]^

**Figure 2:**
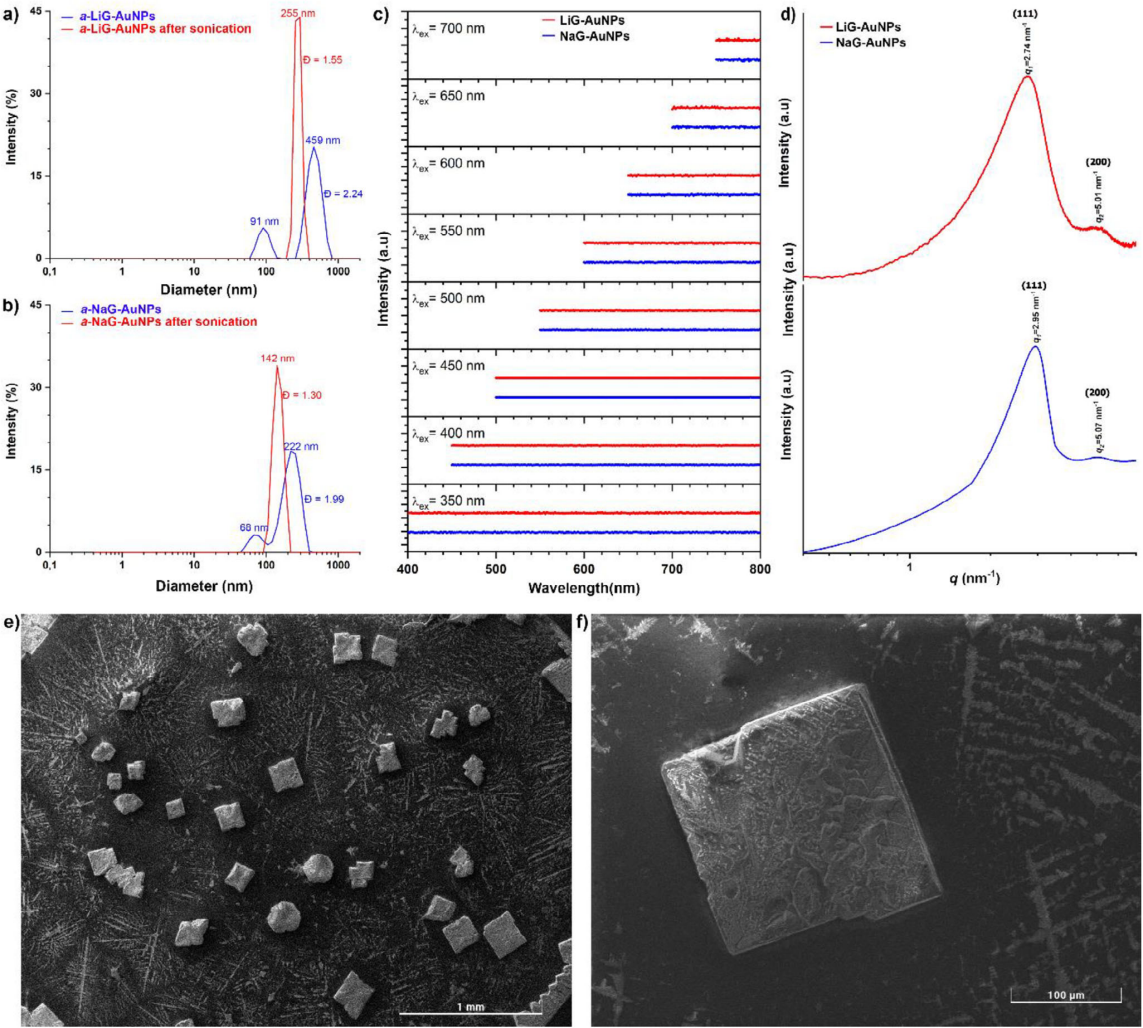
Aggregation tendency of LiG‐AuNPs and NaG‐AuNPs. a) DLS size distribution profiles of *a*‐LiG‐AuNPs and b) *a*‐NaG‐AuNPs water suspensions before and after sonication. c) SAXS patterns of LiG‐AuNPs and NaG‐AuNPs (powder forms). d) Fluorescence profiles of a‐LiG‐AuNPs and a‐NaG‐AuNPs. e,f) SEM micrographs of LiG‐AuNPs (suspended in water, deposed on a glass slide, and dried in air at RT).

Lithium and sodium cations act as crosslinkers for the glutathione‐coated AuNPs. When redispersed in water, both LiG‐AuNPs and NaG‐AuNPs afforded the formation of large aggregates with a broad and multimodal size distribution (blue curves in **Figure** **2**a,b), as verified by dynamic light scattering (DLS) analysis. After sonication (35 kHz, 30 min), the size distribution of the aggregated NPs became narrow and monodispersed (red curves in Figure 2a,b). The colloidal suspension of the *a*‐LiG‐AuNPs was found sufficiently stable in aqueous media, although the size of the aggregates increased to ≈350 and ≈530 nm after 7 and 14 days, respectively.

The aggregation in aqueous suspension affects the photophysical properties of the AuNPs. As a matter of fact, singularly dispersed glutathione‐coated AuNPs present intense excitation at a wavelength of ≈380 nm with corresponding emission in the yellow at a wavelength of ≈575 nm.^[^ ^4^, ^34^ ^]^ Aggregation causes the complete quenching of the luminescence: the emission spectra of *a*‐NaG‐AuNPs and *a*‐LiG‐AuNPs, at variable excitation in the whole UV–Vis spectral width, did not present any emission band (see Figure 2c).

Surprisingly, the aggregation of the NPs in the solid state resulted in an ordered structure, i.e., leading to the formation of a superlattice of NPs. The small‐angle x‐ray scattering (SAXS) analysis of NaG‐AuNPs and LiG‐AuNPs, in powder form, revealed the presence of regularly arranged NPs forming a self‐organized NP array (Figure 2d), i.e., the (111) and (220) SAXS correlation peaks observed were compatible with a face‐centered‐cubic (*fcc*)^[^ ^35^ ^]^ superlattice of AuNPs (see Supporting Information for further details).^[^ ^36^, ^37^, ^38^, ^39^, ^40^, ^41^, ^42^, ^43^ ^]^ The formation of a regular array of NPs was also confirmed by scanning electron microscopy (SEM). The deposition onto a glass slide and the slow drying in air at RT of an aqueous colloidal suspension of LiG‐AuNPs afforded the formation of well‐defined cubic structures consisting of superlattices of NPs with a size of a few hundred micrometers (see Figure 2e,f). The formation of these supramolecular structures of NPs strongly confirms the narrow size distribution of the NPs capable of packing together to form an ordered fcc lattice having an axis *a* ≈4 nm for LiG‐AuNPs and ≈5 nm for the sample NaG‐AuNPs.

Gold and lithium content in LiG‐AuNPs were determined by inductively coupled plasma optical emission spectrometry (ICP‐OES) of acid‐mineralized samples, while the weight percentage of carbon, nitrogen, and sulfur was determined by elemental analysis and found equal to Li = 2.0±0.4%; C = 17.3±0.2%; N = 5.4±0.1%; S = 4.4±0.2% and Au = 53.6±0.3%, approximately corresponding to a molar ratio Li/C/N/S/Au = 1.1:5.3:1.4:0.5/1 and thus to an empirical formula of Li_2_G_1_Au_2_, where all the carboxylate functionalities of the glutathione corona electrostatically interact with lithium cations. This finding agrees with the fact that the synthesis of lithiated nanoparticles has been performed with an excess of lithium hydroxide at a pH above the isoelectric point of glutathione (5.5) and of glutathione attached to the AuNPs (≈ 3.3). Additionally, the precipitation in hydro‐alcoholic media with an excess of lithium chloride affords the complete lithiation of the particles. Considering the average size of 2–3 nm for LiG‐AuNPs, determined by TEM analysis, the most probable clusters with spherical (more precisely with icosahedral morphology) are the NPs with 147 and 309 Au atoms with sizes of 2.02 and 2.59 nm, respectively, derivable from theoretical and geometrical considerations.^[^ ^44^ ^]^ Consequently, the most probable molecular formulas that can be determined for the LiG‐AuNPs are (Li_2_C_10_H_14_N_3_O_6_S)_79_Au_147_ and (Li_2_C_10_H_14_N_3_O_6_S)_166_Au_309_. These molecular formulas compare well with the theoretical ratios between the number of surface atoms and the total number of gold atoms in Au147 and Au309 clusters, respectively, of 92/147 and 162/309, which definitely regulate the effective G/Au molar ratio in the single metallic cluster.

### 2.2 NPs Cytotoxicity, Internalization, and Cell Uptake of Lithium and Gold

**Figure 3:**
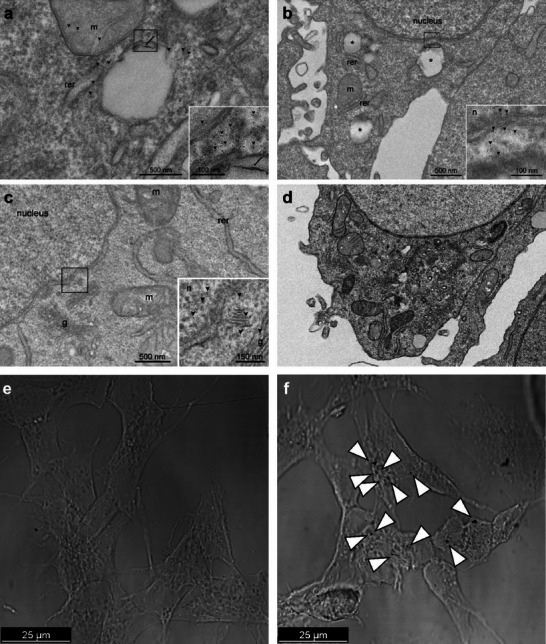
Internalization in the cell of *a*‐NaG‐AuNPs and *a*‐LiG‐AuNPs. a–c) TEM micrographs of Hep G2 cell: i) treated with aqueous suspensions of a‐NaG‐AuNPs (70 µg mL^−1^) for 1 h (m: mitochondria, rer: rough endoplasmic reticulum, g: Golgi apparatus, n: nucleus); ii) d) control experiment in the absence of NPs. AuNPs uptake likely occurred via endocytosis e,f) DIC images of SH‐SY5Y human neuroblastoma cells treated with vehicle (e) and with *a*‐LiG‐AuNPs at 1 mg mL^−1^ concentration for 24 h (panel f; dark spots indicated by arrowheads represent internalized NPs).

The specific physical/structural characteristics of both NaG‐AuNPs and LiG‐AuNPs likely underlie their biological effects. First, as previously demonstrated by Buonerba et al., (2020), *a*‐G‐AuNPs were readily internalized into cells.^[^ ^3^ ^]^ Indeed, TEM analysis confirmed the incorporation of NaG‐AuNPs following HepG2 cell treatment (70 µg mL^−1^ for 1 h) (**Figure** **3**a–c). NaG‐AuNPs and LiG‐AuNPs share several physical/structural characteristics, and indeed, *a*‐LiG‐AuNPs were also rapidly taken up by cells (Figure 3e,f).

This incorporation depended, at least in part, on clathrin‐mediated endocytosis as demonstrated by the use of specific inhibitors of this mechanism, like concavalin A (0.5 µg mL^−1^) and chlorpromazine (10 µg mL^−1^),^[^ ^45^ ^]^ added, separately, to the culture media of SH‐SY5Y cells 4 h before a‐LiG‐AuNP treatment. Indeed, the amount of gold found into the cells was reduced by 75±12% and 46±35% after 1 h of treatment, and 71±24% and 60±13% after 24 h of treatment, respectively (assessed by two‐way ANOVA; p<0.05 for treatment).

Their incorporation into the cell caused the disruption of the aggregates into single nanoparticles. As is known, proteins and other biomolecules present in the cellular media can act as a “corona” for nanoparticles, facilitating the fragmentation of aggregated nanoparticles in biological systems.^[^ ^46^ ^]^ After disaggregation, single NPs were found in the cells' cytoplasm, mitochondria, rough endoplasmic reticulum, Golgi apparatus, and even in the nucleus (Figure 3a–c).

**Figure 4:**
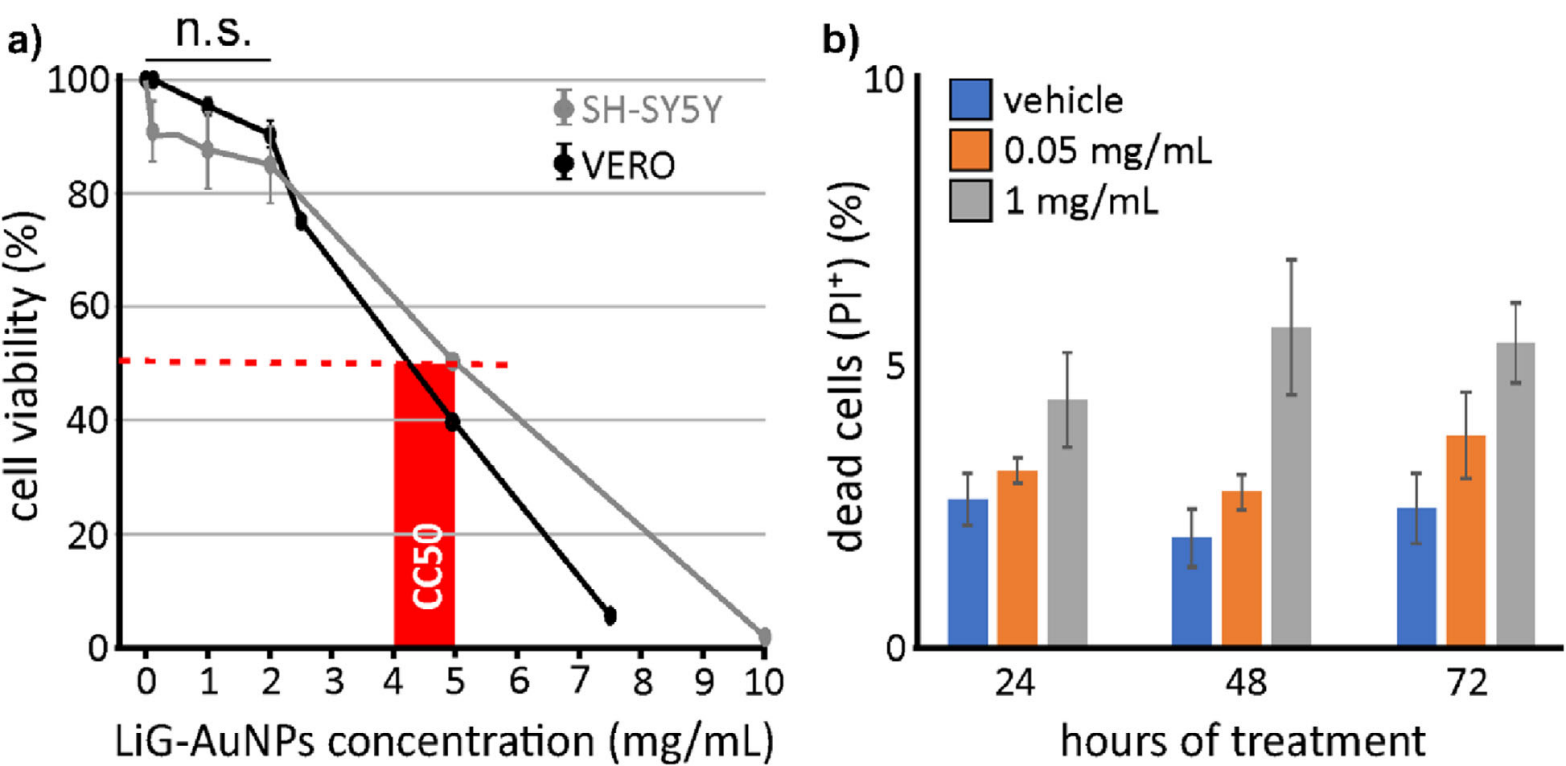
Cell toxicity of *a*‐LiG‐AuNPs. a) Bar graph showing cell viability (% of total) after 24 h of treatment with *a*‐LiG‐AuNPs at various concentrations. NPs were left to aggregate for 1–24 h before being added to the cell culture media. Cell viability was assessed by the Trypan Blue exclusion method, MTT assay, and Propidium iodide (PI) incorporation. b) Bar graph indicating the percentage of the total of SH‐SY5Y cells incorporating PI after 24, 48, and 72 h of incubation with vehicle or *a*‐LiG‐AuNPs at 0.05 and 1 mg mL^−1^. No significant difference was found among treatments.

Based on its ability to internalize as an aggregate and then disaggregate in the cytosol, we tested the efficacy of *a*‐LiG‐AuNPs in modulating GSK‐3β at the cellular level following their incorporation, using in vitro and in vivo experimental models. First, we evaluated the potential toxicity of these nanoparticles to determine the appropriate working concentration for biological samples. To this aim, VERO cells (kidney epithelial cells extracted from an African green monkey) and SH‐SY5Y human neuroblastoma cells were exposed for 24 h to increasing concentrations (0.05–10 mg mL^−1^) of *a*‐LiG‐AuNPs suspended in culture medium. For both experimental models, no significant cytotoxic effects (cell viability ≥ 85%) were observed at concentrations below 2 mg mL^−1^; however, viability significantly decreased at concentrations above this threshold (**Figure** **4**a). The CC50, i.e., the concentration at which 50% of cells die, was estimated to be 4.5–5.0 mg mL^−1^. We then tested the toxicity of longer (72 h) exposure of SH‐SY5Y cells to low *a*‐LiG‐AuNP concentrations (i.e., 0.05 and 1 mg mL^−1^). No significant cell death was observed even at the higher concentration used (<5–10%; Figure 4b).

Once it was demonstrated that LiG‐AuNP aggregates efficiently enter cells and subsequently disaggregate without causing significant cytotoxicity, we investigated their ability to release Li^+^ once dispersed in a cation‐rich medium (e.g., K^+^ and Na^+^). Indeed, the high concentration of cations in the medium could lead to a metal exchange reaction, with Li^+^ electrostatically interacting with the glutathione corona of the NPs, resulting in the release of Li^+^. First, we evaluated the amount of lithium released by *a*‐LiG‐AuNPs after their dispersion in MEM. *a*‐LiG‐AuNPs were sonicated for 5 min in MEM to allow their disaggregation into single NPs, then removed after 1 and 24 h by centrifugal filtration with filters with a molecular weight cut‐off of 5 kDa, and the filtrate was analyzed by ICP‐OES. A lithium content of 0.0017±0.0001 mg/L, 16.71±0.36 mg/L, and 18.28±0.08 mg/L was respectively determined for the untreated MEM and for MEM after 1 and 24 h exposure to the *a*‐LiG‐AuNPs. These findings indicate that nanoparticles efficiently released lithium in such a cation‐containing medium (83.5 and 91.4% of that present on the outer corona after 1 and 24 h, respectively). The supramolecular structure of NPs in LiG‐AuNP aggregates protects internal nanoparticles from releasing lithium when they are in the extracellular, cation‐containing medium, whereas disaggregation into monodispersed particles following internalization allows for efficient lithium delivery.

We further performed experiments aimed at determining if aggregates protect internal lithium from being released by cation exchange. To this end, LiG‐AuNP aggregates were incubated in two different media: deionized water and 3 mM NaCl (in water). The NaCl suspension was then subjected to sonication to disrupt larger aggregates and homogenize aggregate size distribution, thereby enabling lithium release from previously shielded inner regions.

Phase separation between the solid and liquid fractions (which include ions detached from AuNPs and dissolved in solution) was achieved using dedicated filtration columns, and lithium content was quantified in both phases under each condition. In bi‐distilled water, LiG‐AuNPs lost ≈33.4±6.6% of their lithium content 1 h after being dissolved. In the NaCl medium, the percentage of lithium released was, as expected, higher (65.0±6.2% of the total load, lower than that found in culture media), consistent with increased cation exchange. Nevertheless, sonication led to a further ≈5% increase in lithium concentration in the liquid phase compared to the non‐sonicated solution (69.8±4.4%). Correspondingly, the amount of lithium retained in the solid fraction was reduced in sonicated LiG‐AuNPs with respect to the non‐sonicated counterpart (∼ −7%). Although the absolute difference is modest, it is essential to consider that sonication does not entirely disrupt aggregates but instead reduces heterogeneity by fragmenting larger structures, thereby homogenizing all aggregates to ≈250 nm in diameter. This action enables the selective release of lithium from larger aggregates (>250 nm) that are disrupted. We then employed an experimental paradigm that allowed for partial disruption of aggregates, reducing their diameter, as demonstrated by the addition of glucose (10 mg/mL) and BSA (1 mg mL^−1^), as shown by DLS measurements (Figure S1a,b, Supporting Information). In this condition, we found that the amount of lithium released was ≈45% higher in water (48.9±1.9%; p = 0.03) and 7% higher in NaCl respect to the sonicated condition without glucose and BSA (Figure S1c, Supporting Information), thus supporting our hypothesis that aggregates protect inner lithium to be released in solution.

Next, we determined the efficiency of *a*‐LiG‐AuNPs in conveying lithium cations into cells. To this aim, human neuroblastoma SH‐SY5Y cells were incubated with *a*‐LiG‐AuNPs (1 mg_/_mL, corresponding to 3 mmol_Li+_/L or mEq_Li+_/L) for 24 h, and the amount of lithium incorporated into the cells was determined by ICP‐OES analysis. For comparison, cells were otherwise treated with LiCl at the same concentration of extracellular lithium (3 mmol_Li+_/L or 3 mEq_Li+_/L). Notably, cells do not have ion channels selective for Li^+^, and this cation enters cells mainly via Na^+^ channels.^[^ ^47^ ^]^ Li^+^ concentration found in LiG‐AuNP‐treated cells was ≈26.5 times higher (in a range of 0.154–0.337 pg cell^−1^) than that found in LiCl‐treated cells (range: 0.007–0.037 pg_Li_/cell), confirming that the former is a more efficient shuttle of this metal cation into the cell, producing a higher intracellular concentration under comparable experimental conditions. The corresponding uptake of gold in SH‐SY5Y cells was in the range 3.8–17.2 pg_Au_/cell. As a reference, previous experiments of incorporating *a*‐NaG‐AuNPs in HepG2 cells afforded gold incorporation of 2.6±1.2 pg_Au_/cell.^[^ ^3^ ^]^

### 2.3 LiG‐AuNP‐Induced Inhibition of GSK‐3β Via Phosphorylation at Ser9 in in Vitro and ex‐Vivo Models

#### 2.3.1 In Vitro Investigations

**Figure 5:**
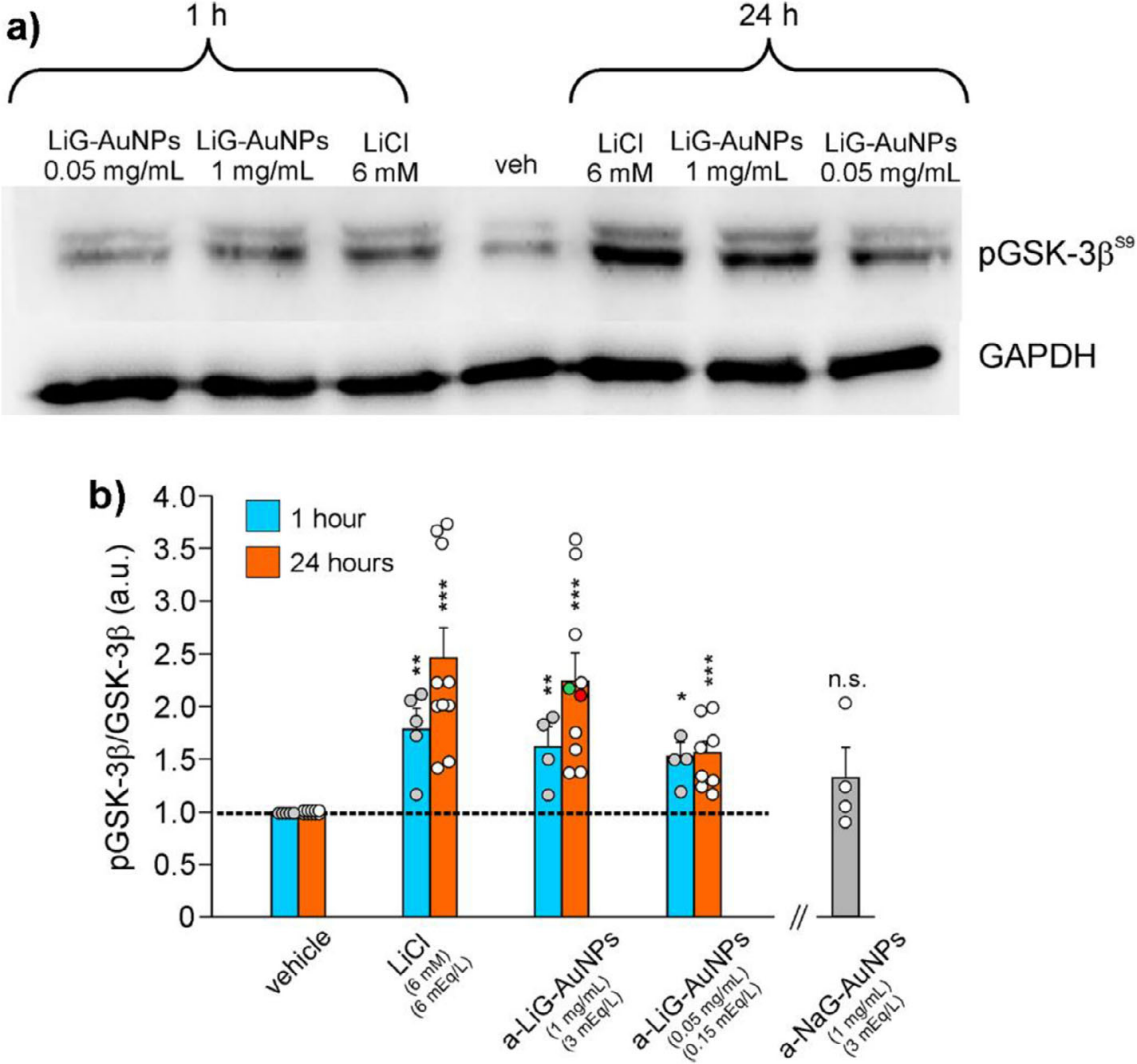
LiG‐AuNPs induce phosphorylation of GSK‐3β at Ser9 in cultured cells. a) Representative WB analysis of pGSK‐3β^S9^ carried out on lysates of human neuroblastoma cells SH‐SY5Y treated for 1 and 24 h with LiCl (6 mM, equal to 6 mEq L^−1^ of Li^+^) and *a*‐LiG‐AuNPs at 1 mg/mL (3 mEq L^−1^ of Li^+^) and 0.05 mg/mL (0.15 mEq L^−1^ of Li^+^), left to aggregate for 1–7 days in deionized water at +4 °C (GAPDH was used as loading control). b) Bar graph quantifying experiments in panel (a). The bar on the right (grey) represents the quantification of WB analysis carried out on lysates of SH‐SY5Y cells treated for 24 h with 1 mg mL^−1^ aggregates of NaG‐AuNPs. Red and green dots in the distribution of *a*‐LiG‐AuNPs (1 mg mL^−1^, 24 h) represent values obtained in A549 cells and murine astrocytes, respectively. **p<0.01 versus vehicle; ***p<0.001 versus vehicle. n.s. indicate no significant difference versus vehicle. Statistical significance was assessed by an ANOVA test with Tukey *post‐hoc* correction.

Given the high efficiency of *a*‐LiG‐AuNPs in delivering lithium into cells, we evaluated their ability to induce the inhibitory phosphorylation of GSK‐3β at Ser9. To this aim, SH‐SY5Y cells were treated for either 1 h or 24 h with *a*‐LiG‐AuNPs at a concentration of 1 mg mL^−1^ (3 mEq_Li+_/L). As a positive control, cells were incubated in a culture medium containing 6 mM LiCl (6 mEq_Li+_/L) for both 1 and 24 h. This LiCl concentration was chosen because it represents the lowest concentration commonly used in in vitro studies to induce GSK‐3β inhibition.^[^ ^26^ ^]^ As expected, 6 mM LiCl significantly increased pGSK‐3β^Ser9^. By assuming equal to “1” the ratio between phosphorylated (pGSK‐3β^Ser9^) and total GSK‐3β (pGSK‐3β^Ser9^/GSK‐3β) in control, vehicle‐treated cells (n = 6 and n = 13 independent experiments for 1‐h and 24‐h‐lasting treatments, respectively), after cell exposure to 6 mM LiCl this ratio raised to 1.78±0.25 (n = 4; p = 0.019 vs control) and 2.45±0.29 (n = 10; p<1×10^−3^ vs control) for 1 and 24 h treatments, respectively (**Figure** **5**). Surprisingly, Li^+^ exerted similar effects at half concentration when applied via LiG‐AuNPs. Indeed, by using *a*‐LiG‐AuNPs at the safe concentration of 1 mg/mL, corresponding to 3 mEq/L Li^+^, pGSK‐3β^Ser9^/GSK‐3β values were: i) 1.60±0.20 (n = 4 independent experiments; p = 0.048 vs vehicle; not significant vs 6 mM LiCl) following 1‐h treatment; ii) 2.23±0.26 (n = 10; p<1×10^−3^ vs vehicle; not significant vs 6 mM LiCl) following 24‐h treatment (Figure 5). We also checked the ability of very low lithium concentrations (0.05 mg/mL, corresponding to 0.15 mEq_Li_/L) delivered via *a*‐LiG‐AuNPs to inhibit GSK‐3β. Following 1 and 24 h treatments the pGSK‐3β^Ser9^/GSK‐3β ratio were 1.47±0.13 (n = 4; p = 0.023 vs vehicle) and 1.54±0.12 (n = 8; p<1×10^−3^ vs vehicle), respectively (Figure 5). Instead, an equal amount of LiCl (0.15 mM) did not exert any significant effect (pGSK‐3β^Ser9^/GSK‐3β = 1.14±0.14; n = 4; not significant ‐p = 0.495‐ vs vehicle after 24‐h treatment; data not shown).

These data indicate that phosphorylation levels of GSK‐3β at Ser9 correlate with the intracellular concentration of lithium obtained by cell treatment with *a*‐LiG‐AuNPs. Notably, the effect of *a*‐LiG‐AuNPs on pGSK‐3β^Ser9^ did not depend on the nature of cells used, being virtually SH‐SY5Y neuroblastoma cells, immortalized lung cells (A549), and cultured murine cortical astrocytes (Figure 5). Finally, to address the specificity of Li^+^ effects on pGSK‐3β and exclude the non‐specific action of AuNPs, we investigated the effects of 24‐h treatment with *a*‐NaG‐AuNPs on pGSK‐3β^Ser9^ levels. As expected, no significant differences in pGSK‐3β^Ser9^/GSK‐3β levels (1.32±0.29, n = 4; p = 0.794 vs vehicle) were observed in cells treated for 24 h with *a*‐NaG‐AuNPs in the same conditions of treatments with *a*‐LiG‐AuNPs (Figure 5), thus suggesting that the observed effects are specifically due to lithium delivered into the cell by the nanocarrier.

#### 2.3.2 Intranasal Administration of a‐LiG‐AuNPs Induces Inhibition of GSK‐3β by Phosphorylation at Ser9 Within the Hippocampus of Living Mice

The study of the inhibitory effects of *a*‐LiG‐AuNPs on GSK‐3β activity was thus extended to in vivo models. One of the main goals of researchers working on GSK‐3 is to modulate GSK‐3β activity in specific target organs (e.g., brain or lungs), given the involvement of this kinase in many neurological diseases, including mood disorders, tauopathies, Alzheimer's disease, Parkinson's disease, viral encephalitis, or in diseases of the respiratory tract due to viral infections such as COVID‐19. However, while lungs can be easily reached by aerosol, direct access to the brain is more critical. In psychiatric disorders, lithium is taken up by oral administration of lithium carbonate that is distributed to the whole body through circulation, thus also reaching organs that are highly susceptible to the toxic action of the ion, such as the thyroid and kidneys. We exploited our nanocarriers for a targeted Li^+^ delivery to the brain via intranasal administration of *a*‐LiG‐AuNPs in adult C57BL/6 mice. Then, pGSK‐3β^Ser9^ levels were measured in different cerebral areas, namely the hippocampus, neocortex, and olfactory bulb.

**Figure 6:**
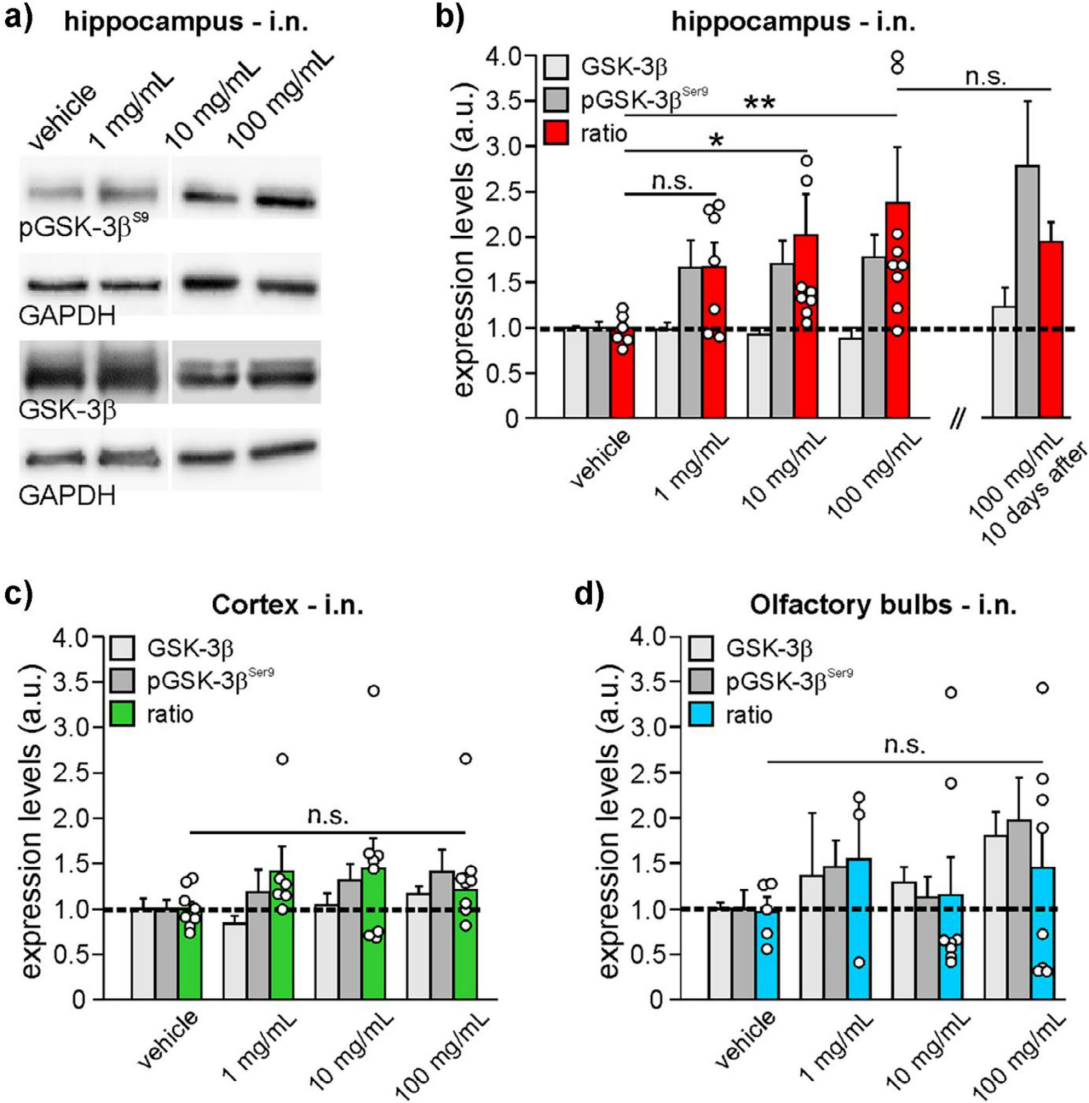
Intranasal administration of *a*‐LiG‐AuNPs modulates phosphorylation of GSK‐3β at Ser9 in the hippocampus of adult mice. a) Representative WB analysis of hippocampal tissue obtained from mice treated for 5 consecutive days with *a*‐LiG‐AuNPs at various concentrations (and left to aggregate for 24 h in deionized water), administered intranasally. Lysates were probed with antibodies against pGSK‐3β^Ser9^ and anti‐GSK‐3β (total). GAPDH was used as a loading control (Lanes on the left vehicle and 1 mg/mL have been spliced with lanes 10–100 mg from the same gel). b) Bar graph showing the expression levels of total GSK‐3β, pGSK‐3β^Ser9^, and their ratio (pGSK‐3β^Ser9^/GSK‐3β; red bars) of the condition represented in panel (a). The dotted line represents values of control (vehicle, = 1); c,d) Bar graph showing the expression levels of total GSK‐3β, pGSK‐3β^Ser9^, and their ratio (green and blue bars), for cortices (c) and olfactory bulbs (d) of mice treated as in (a). * p<0.05, ** p<0.01, n.s. means not a significant difference.

Mice were treated for 5 consecutive days with *a*‐LiG‐AuNPs dissolved in deionized water at various concentrations (0, 1, 10, and 100 mg mL^−1^). For intranasal administration: 3 µL/nostril/day (Figure S2, Supporting Information). These concentrations were chosen based on in vitro results and information on lithium doses commonly used to initiate therapy in patients (≈0.0023 mg_Li_ × g of body weight/day, corresponding to ≈0.012 mg_Li_/g for 5 days).^[^ ^48^ ^]^ By assuming that *a*‐LiG‐AuNPs contain ≈2 *wt*% of lithium, the maximum tested concentration (6 µL at 100 mg/mL × 5 days) corresponds to ≈0.012 mg_Li_/g of mouse body weight. 6 h after the last administration of *a*‐LiG‐AuNPs (either intranasal or oral), mice were sacrificed, the brain was removed, and then divided into two parts by sagittal cutting. One hemisphere was sectioned to isolate different areas (hippocampus, neocortex, olfactory bulb) and then processed for WB, whereas the contralateral one was used to quantify the amount of gold and/or lithium reaching the brain by ICP‐OES. Our analyses indicated that intranasal administration of *a*‐LiG‐AuNPs was able to modulate GSK‐3β activity in the brain, especially in the hippocampus. In this area, we found that *a*‐LiG‐AuNPs did not significantly affect the expression of “total” GSK‐3β at any concentration tested (p = 0.239). On the contrary, the pGSK‐3β^Ser9^/GSK‐3β ratio increased in dose‐dependent manner with the concentration of *a*‐LiG‐AuNPs applied, passing from 1.00±0.06 (vehicle; n = 8 brains), to 1.67±0.27 (1 mg mL^−1^, n = 7; p = 0.083 vs vehicle), 2.02±0.45 (10 mg/mL, n = 9; p = 0.016 vs vehicle), and 2.37±0.62 (100 mg mL^−1^, n = 9; p = 0.006 vs vehicle) (**Figure** **6**a,b). Despite the increase in pGSK‐3β^Ser9^ levels, no significant modification of gold content was found in the brains of *a*‐LiG‐AuNP‐treated mice. Indeed, in vehicle‐treated brains, a gold content equal to 6.7±1.3 ng/mg_hemisphere_ (n = 19 hemispheres) was determined by ICP‐OES, whereas in the brain of mice treated with either 10 or 100 mg/mL *a*‐LiG‐AuNPs, the mean values of gold were 8.7±1.4 ng/mg_hemisphere_ (n = 10 hemispheres) and 6.1±1.5 ng/mg_hemisphere_ (n = 11 hemispheres), respectively (p = 0.45 by one‐way ANOVA test). It was not possible to quantify lithium because its levels were below the detection threshold of the used equipment.

Interestingly, in mice sacrificed 10 days after the last intranasal administration of 100 mg mL^−1^ LiG‐AuNPs, pGSK‐3β^Ser9^ was yet higher than control, being 2.19 ± 0.25 (n = 4 mice; p = 1.1×10^−4^ vs vehicle, not significant vs 100 mg/mL evaluated 6 h after sacrifice; Figure 6b) thus indicating that the effects of *a*‐LiG‐AuNPs last several days after the end of treatment.

On the contrary, intranasal administration of LiCl at lithium concentrations equivalent to that present in 100 mg mL^−1^ LiG‐AuNPs for five consecutive days did not induce any significant effects in the ratio pGSK‐3β^Ser9^/GSK‐3β at the hippocampal level (1.24±0.08 vs 1.00±0.08 of vehicle; n = 3 mice for conditions, p>0.05; data not shown).

The modulation of GSK‐3β activity observed in the neocortices and olfactory bulbs of treated mice was lower than in the hippocampi, and it was not dose‐dependent. Indeed, the treatment with *a*‐LiG‐AuNPs (1, 10, 100 mg mL^−1^) did not change the expression of either total GSK‐3β (p = 0.07 and p = 0.222, respectively) or the pGSK‐3β^Ser9^/GSK‐3β ratio (p = 0.424 and p = 0.958, respectively). Indeed, by setting to “1” the levels of pGSK‐3β^Ser9^/GSK‐3β in the neocortices of mice treated with vehicle only, the other values were: 1.42±0.27 (for 1 mg mL^−1^; n = 6 independent brains), 1.45±0.34 (for 10 mg mL^−1^; n = 7) and 1.13±0.19 (for 100 mg mL^−1^; n = 8) (Figure 6c). For the olfactory bulbs, the values were: 1.56±0.71 (for 1 mg mL^−1^; n = 3 independent brains), 1.15±0.42 (10 mg mL^−1^, n = 8), and 1.45±0.45 (100 mg mL^−1^, n = 8) (Figure 6d).

These results indicate that intranasal administration of LiG‐AuNPs is an effective and controlled method for delivering lithium ions to the brain, particularly to the hippocampus.

#### 2.3.3 Long‐Term Administration of a‐LiG‐AuNPs Modulates Hippocampal GSK‐3β Without Affecting the Health Status of Mice

**Figure 7:**
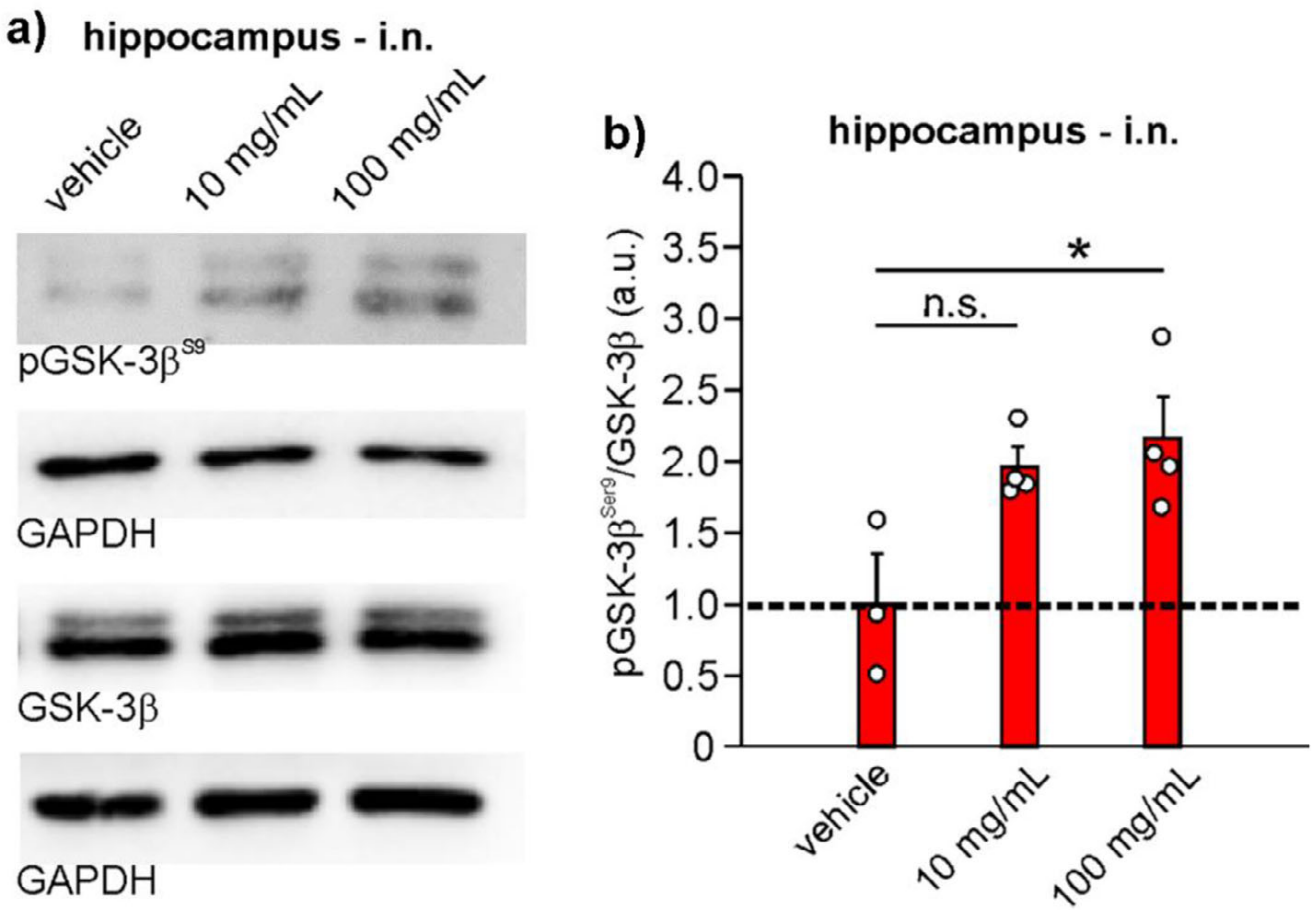
Intranasal administration of *a*‐LiG‐AuNPs for 5 months modulates phosphorylation of GSK‐3β at Ser9 in the hippocampus of adult mice. a) Representative WB analysis of hippocampal tissue obtained from mice treated with *a*‐LiG‐AuNPs at various concentrations for 5 months (as in Figure S1, Supporting Information), administered intranasally. Lysates were probed with antibodies against pGSK‐3β^Ser9^ and anti‐GSK‐3β (total). GAPDH was used as a loading control. b) Bar graph showing the expression levels of the ratio (pGSK‐3β^Ser9^/GSK‐3β; red bars) of the condition represented in panel (a). * p<0.05, n.s. means no significant difference.

It is known that GSK‐3 hyperactivation plays a crucial role in the aetiology of many neurodegenerative and neuropsychiatric illnesses, which often persist for months or even years. Given the ability of *a*‐LiG‐AuNPs to induce inhibitory phosphorylation at Ser9 after a 5‐day treatment, we aim to investigate the potential use of *a*‐LiG‐AuNPs for chronic, long‐lasting treatment. Specifically, the intranasal administration of *a*‐LiG‐AuNPs was performed once daily for 5 consecutive days, and this protocol was repeated every other week (i.e., twice a month) for 5 months (see Supplementary Figure 1). Given its low efficacy in short‐term treatments, the 1 mg mL^−1^ dose was excluded from the investigation, and doses of 10 and 100 mg mL^−1^ were tested. The choice to treat mice for 5 days with an interval of 10 days stems from the observation that 10 days after the last administration of *a‐*LiG‐AuNPs, pGSK‐3β levels were still high. Therefore, we hypothesized that with this protocol, GSK‐3β was continuously inhibited. This hypothesis was verified by the observation that no significant differences in pGSK‐3β^Ser9^ were found at the end of the long‐term treatment compared to the short‐term one. Indeed, the ratio pGSK‐3β^Ser9^/GSK‐3β was 1.96±0.14 and 2.14±0.30, respectively, for 10 and 100 mg mL^−1^, by considering equal to 1 the mean value of pGSK‐3β of the vehicles (p = 0.039 and p = 0.017, respectively vs vehicle; **Figure** **7**a,b). We then wondered if long‐term administration resulted in the accumulation of gold in the brains of treated mice. We found that in mice treated with the lowest dose, the amount of gold in the brain was estimated at 4.0±1.1 ng/mg_hemisphere_ (n = 4 hemispheres), whereas the highest dose determined a modest but significant accumulation of gold (23.1±7.0 ng/mg_hemisphere_; n = 4 hemispheres), as determined by ICP‐OES analysis.

The presence of gold in the brain could trigger a defensive response by astrocytes and microglia, which act as immunocompetent cells within the brain, leading to astrogliosis, an unquestionable sign of neuroinflammation. Following activation, astrocytes express enhanced levels of glial fibrillary acidic protein (GFAP), which is a marker protein for astrogliosis; GFAP levels in the brain of mice intranasally treated with *a*‐LiG‐AuNPs were thus investigated by WB analysis. No significant differences in GFAP levels were observed among the various treatments and the vehicle, even when *a*‐LiG‐AuNPs were administered continuously for five months (Figure S3; p = 0.165, Supporting Information), indicating no astrogliosis in the hippocampus of the mice. Moreover, according to the current legislation about the assessment of the well‐being of laboratory mice, we also monitored the general state of health of the mice by evaluating the following parameters throughout the duration of the long‐term treatment with *a*‐LiG‐AuNPs: state of the coat (that must be shiny, smooth, sleek), weight, and the occurrence of abnormal behavior reflecting signs of sickness such as apathy: we did not observe any significant anomalies (see Figure S4a,b, Supporting Information).

Several previous reports indicate that nanoparticles, including AuNPs, are efficiently eliminated from the brain through the glymphatic system.^[^ ^49^, ^50^ ^]^ Zhang et al., in 2012, demonstrated that GSH‐protected AuNPs can be efficiently metabolized by renal clearance.^[^ ^51^ ^]^ We then measured the level of gold in the kidneys of a‐LiG‐AuNP‐treated mice, which were administered for 2 months (following our experimental paradigm of 5 days of treatment followed by 10 days of rest, repeated cyclically), and correlated it with the amount of gold in the brains of the same animals. Our results showed a positive correlation (m = 0.280; p = 0.001) between the two measures, thus indicating that gold is (likely) eliminated by the urinary system (Figure S4c, Supporting Information).

#### 2.3.4 Intranasal Treatment With a‐LiG‐AuNPs Does not Alter Plasma Lithium Levels in Mice

**Figure 8:**
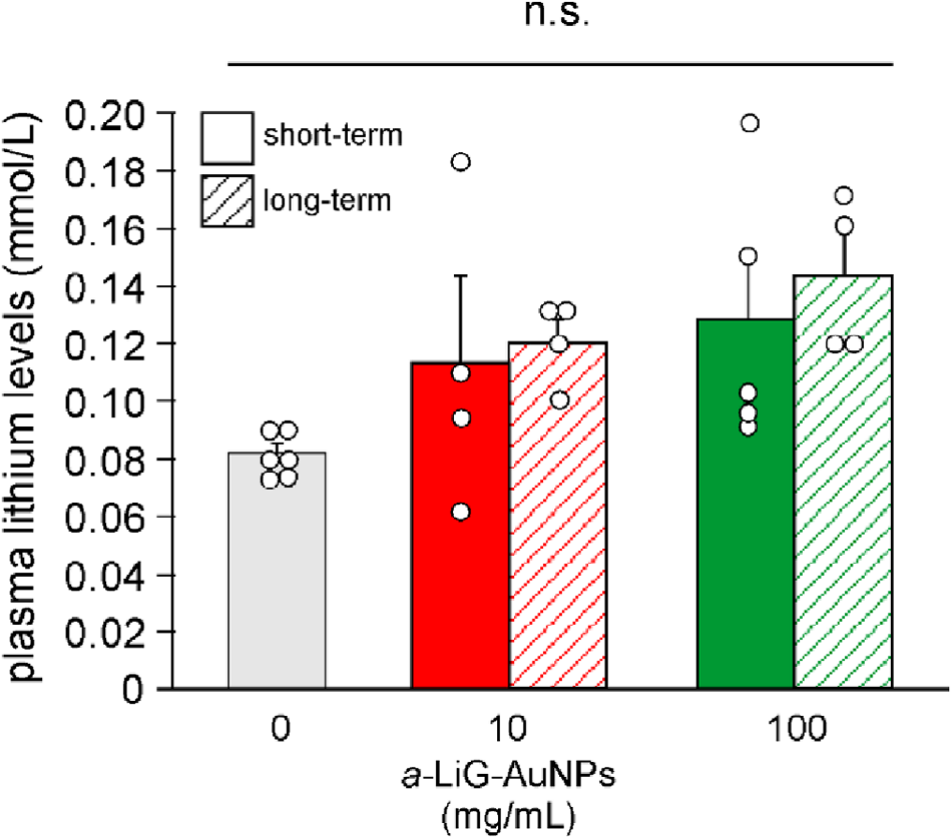
Intranasal administration of *a*‐LiG‐AuNPs does not alter plasma lithium levels in mice. Bar graph quantifying lithium levels (mmol/L) in plasma samples of mice treated both for only 5 consecutive days with *a*‐LiG‐AuNPs administered either intranasally or by oral gavage (grey, red, and green bars) and for a longer time (as in Figure S2, Supporting Information) (pink bar). Plasma samples were obtained immediately before the mice were sacrificed. ** p<0.01 versus vehicle; n.s. means no significant difference versus vehicle assessed by Student's t‐test.

Lastly, we wondered if the *a*‐LiG‐AuNPs treatment altered the amount of lithium ions present in the plasma of mice. We then collected blood from vehicle‐ and NP‐treated mice (both 10–100 mg mL^−1^ *a*‐LiG‐AuNPs) before sacrifice and then analyzed the blood samples to quantify lithium levels in the plasma. After the five‐day administration, we found that vehicle‐treated mice had basal plasma lithium levels equal to 0.082±0.003 mmol L^−1^ (n = 6 independent samples of plasma from treated mice), whereas *a*‐LiG‐AuNP‐intranasally‐treated ones exhibited values equal to 0.113±0.030 mmol/L for 10 mg mL^−1^‐treated mice (n = 4 plasma samples) and 0.128±0.023 mmol L^−1^ for 100 mg/mL‐treated ones (n = 5; **Figure** **8**).

In mice that were intranasally treated with *a*‐LiG‐AuNPs for 5 months, plasma lithium levels measured before sacrifice were still not significantly different from those of control mice (0.120±0.008 mmol L^−1^, n = 4, and 0.143±0.015 mmol L^−1^, n = 4, for 10 and 100 mg mL^−1^ treated mice, respectively; Figure 8). This suggests that intranasal administration of LiG‐AuNPs does not alter plasma lithium levels, even after prolonged treatment.

#### 2.3.5 a‐LiG‐AuNPs Treatment Reduced tau Protein Phosphorylation and Ameliorated Memory Performance in old AD Mouse Models

**Figure 9:**
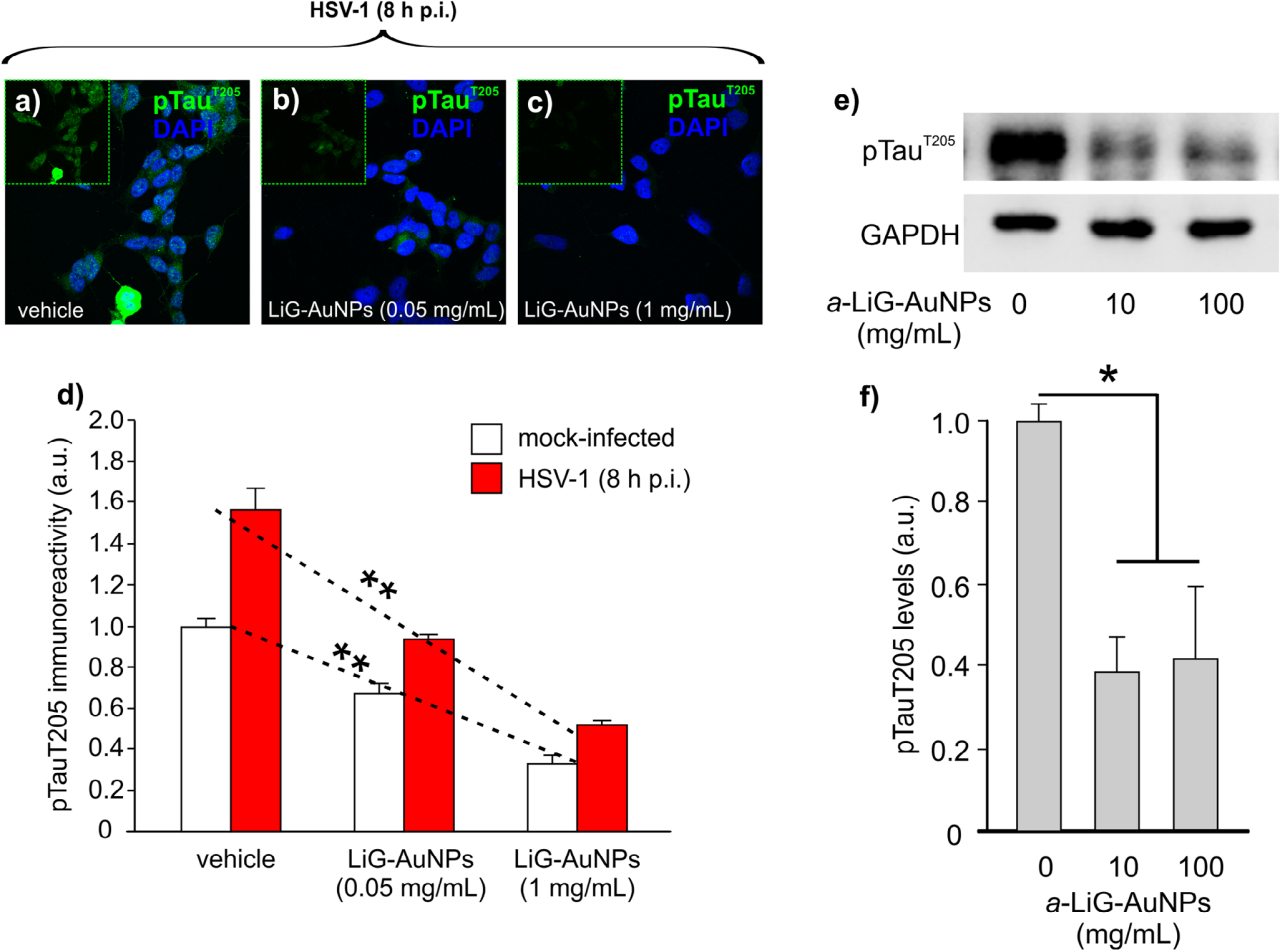
*a*‐LiG‐AuNP treatment determines the reduction of tau phosphorylation at Thr205 in cultured cells and mouse brains. a–c) Representative images of SH‐SY5Y human neuroblastoma cells treated for 24 h with vehicle (a) or *a*‐LiG‐AuNPs (left to aggregate for 1 day) at a concentration of 0.05 mg mL^−1^ (b) and 1 mg mL^−1^ (c). Boxes in the upper left of the panels (a–c) represent IF images of pTau^Thr205^ only. Cell nuclei were labeled blue by DAPI staining. d) Bar graph showing quantification of pTau^Thr205^ in the conditions represented in (a–c). e) Representative WB analysis of hippocampal tissue obtained from mice treated with *a*‐LiG‐AuNPs at various concentrations for 5 months (as in Figure S1, Supporting Information), administered intranasally. Lysates were probed with antibodies against pTau^Thr205^. GAPDH was used as a loading control. f) Bar graph showing the quantification of the experiments reported in panel (e). * p<0.05; ** p<0.01. Statistical significance was assessed by the One‐Way ANOVA test followed by Bonferroni *post‐hoc* correction.

The effect of GSK‐3β inhibition on the phosphorylation of its substates was also studied. Considering its important role in Alzheimer's disease pathophysiology, the phosphorylation of tau protein at Thr205 was investigated in both in vitro and in vivo models by IF and WB experiments. For in vitro studies, we exploited the experimental paradigm of tau hyperphosphorylation induced by Herpes Simplex virus type 1 infection.^[^ ^13^ ^]^ SH‐SY5Y human neuroblastoma cells were thus infected or not with HSV‐1 and treated with *a*‐LiG‐AuNPs used at 0.05 and 1 mg/mL for 24 h, then fixed in formalin solution 10% and finally processed for IF. As shown in **Figure** **9**a–d, the treatment with *a*‐LiG‐AuNPs significantly reduced pTau^Thr205^ in both conditions and concentrations tested. Notably, we also found that in the hippocampus of long‐term‐treated mice, *a‐*LiG‐AuNPs reduced the “basal” pTau^T205^ levels (Figure 9e,f), consistent with results reported for in vitro experiments.

**Figure 10:**
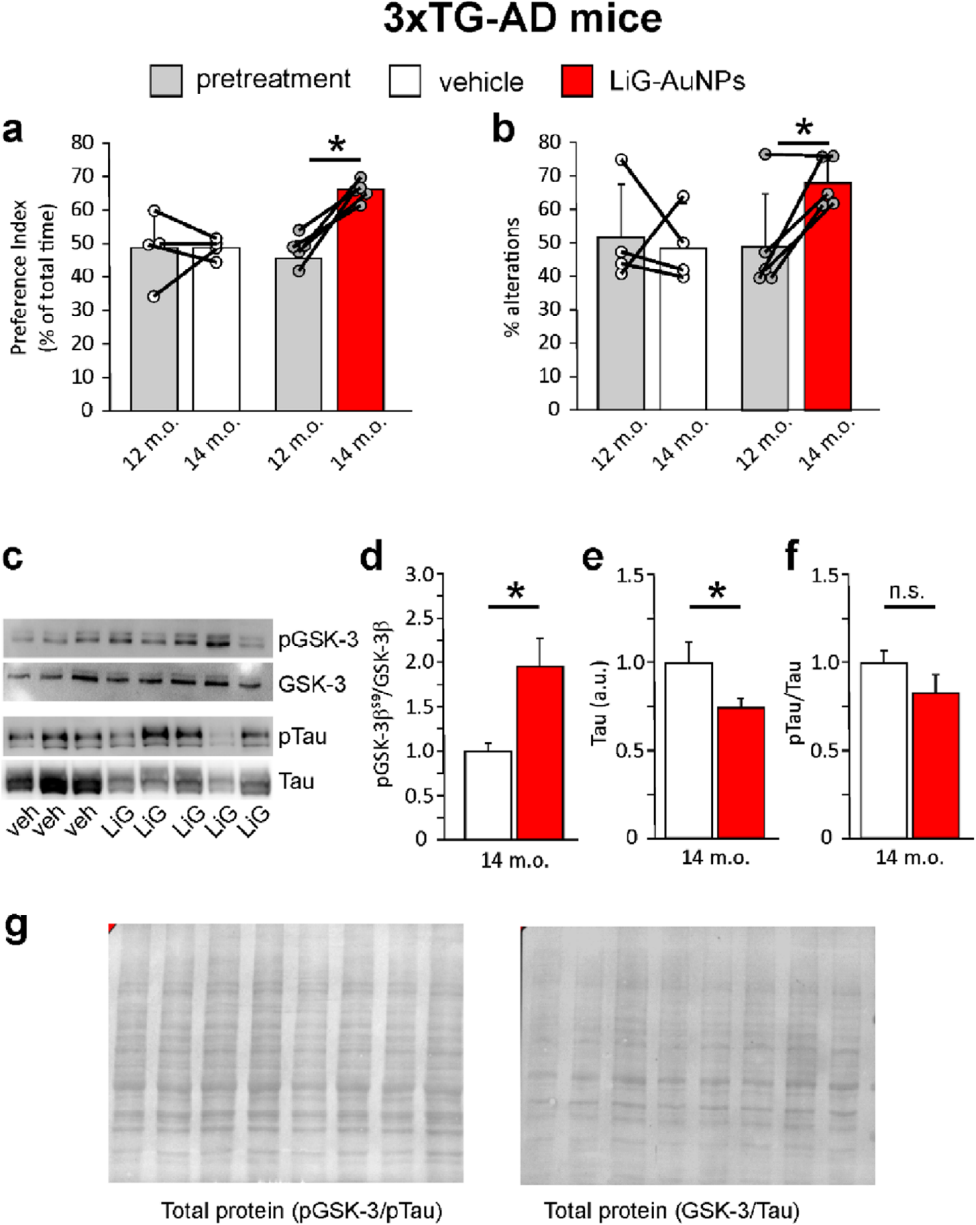
*a*‐LiG‐AuNP treatment ameliorates memory deficits in 3×Tg‐AD mice. a) Bar graph showing the preference indices evaluated during the NOR paradigm of 3×Tg‐AD mice, treated with either vehicle or *a*‐LiG‐AuNPs (3 µL/nostril of 100 mg mL^−1^ stock solution) starting at 12 months of age (pretreatment, grey bars) until 14 months (white ‐vehicle; red – *a*‐LiG‐AuNPs). b) Bar graph showing the number of alternations during the Y‐maze paradigm of 3×Tg‐AD mice, treated as in (a). c) Representative WB analysis of hippocampal tissue obtained from mice treated as in (a and b) at the end of vehicle and *a*‐LiG‐AuNP treatment. Lysates were probed with antibodies against pGSK‐3β^Ser9^, GSK‐3β, pTau^Ser396,^ and Tau13 (total Tau). WBs were normalized to the total amount of proteins. d) Bar graph showing the ratio of pGSK‐3β^Ser9^ and GSK‐3β in the hippocampal tissue of 14‐month‐old mice as in (c). e,f) Bar graphs showing the quantification of total tau and the ratio pTau/Tau, respectively. g) Representative examples of total protein used for normalization for GSK‐3 and tau. * means p<0.05.

We then aimed at demonstrating the potential beneficial effects of a‐LiG‐AuNP treatment against memory decline occurring in the 3×Tg‐AD mouse model of Alzheimer's disease.^[^ ^52^ ^]^ We selected 12‐month‐old mice that exhibit strong memory deficits at this age (see additionally: https://www.alzforum.org/research‐models/3xtg), as assessed by the novel object recognition (NOR) and Y‐maze tests. We divided the mice into two groups: one receiving only vehicle (water) and the other treated with *a*‐LiG‐AuNPs. We treated both groups for two months, following the protocol that yielded the best results in pGSK‐3β modulation after prolonged intranasal administration. Specifically, treatment consisted of five consecutive days of administration (either water or LiG‐AuNPs), followed by a 10‐day recovery period, repeated cyclically. At the end of LiG‐AuNP treatment, AD mice exhibited a significant improvement in memory performance, which was not observed in vehicle‐treated ones. In the NOR test, the preference index increased from 45.4±3.9 before treatment to 65.9±1.6 (n = 5 mice; p<0.001; **Figure** **10**a) one week after the last dose. Conversely, vehicle‐treated mice showed no significant changes over time (48.7±6.0 vs 48.6±1.7; Figure 10a). Similarly, in the Y‐maze test, the number of alternations increased from 49.4±7.8 before treatment to 68.2±3.7 (p<0.05; Figure 10b) in the *a*‐LiG‐AuNP‐treated group, while no improvement was seen in vehicle‐treated mice (51.8±9.1 vs 48.8±6.3; Figure 10a). At the molecular level, memory improvement in *a*‐LiG‐AuNP‐treated mice was associated with a significant increase in pGSK‐3β^Ser9^ in the brain (Figure 10c) and a significant reduction of total tau (‐48%; p = 0.010; Figure 10d–f), along with a proportional decrease of pTau (Figure 10e,f).

Furthermore, to assess whether these effects could be attributed to a specific action on proteins involved in synaptic communication, we evaluated whether *a*‐LiG‐AuNP treatment could prevent synaptic protein downregulation induced by extracellular tau oligomers (oTau) in primary hippocampal neuron cultures. Notably, in cells treated with *a*‐LiG‐AuNPs (at a very low lithium concentration of 0.15 mEq L^−1^), oTau (200 nM, 1 h) failed to reduce synapsin‐1 expression.^[^ ^53^ ^]^ This protective effect was not observed in neurons treated for the same duration with LiCl at the same lithium concentration (Figure S5, Supporting Information).

## 3 Discussion

Lithium, apparently a simple metal cation, has important pharmacological applications. It is a potent inhibitor of the hub kinase GSK‐3β, whose β isoform (GSK‐3β) is involved in the development of a plethora of illnesses, including mood disorders (e.g., bipolar disorder), neurodegenerative diseases (e.g., tauopathies and Alzheimer's disease), and virus‐dependent infectious diseases (HSV‐1 infection, coronaviruses ‐e.g., SARS‐CoV‐1 and SARS‐CoV‐2‐mediated infections, etc.).^[^ ^18^ ^]^ Indeed, its therapeutic effects in mood disorders,^[^ ^54^ ^]^ viral infections,^[^ ^18^ ^]^ and neurodegenerative diseases^[^ ^55^ ^]^ have been extensively recognized. Unfortunately, the narrow window of useful concentrations dramatically limits its application. Indeed, although the use of lithium in clinical practice dates back to 1870, especially for the treatment of psychiatric disorders,^[^ ^21^ ^]^ it cannot be taken without risks, given that it is highly toxic (up to being lethal) for humans, especially affecting the kidneys and thyroid. For this reason, patients taking lithium (exclusively by oral administration) must regularly monitor plasma lithium levels, which cannot go beyond 0.8–1.2 mEq L^−1^, and not all patients (e.g., elderly persons) can take lithium without risks. Moreover, lithium concentrations suitable for treating psychiatric disorders are often too low to contrast other GSK‐3β‐dependent illnesses, such as neurodegenerative diseases or viral infections.^[^ ^56^ ^]^ Therefore, organ‐specific lithium‐releasing devices that ensure an efficient release of the cation and overcome the side effects due to high‐concentration therapeutic doses would be highly desirable.

The high ion mobility of lithium also contributes to the challenge of developing methods for its targeted delivery in the human body. Normal cell control systems also hinder their penetration into the cell membrane. This study reports the synthesis and characterization of gold nanoparticles (AuNPs) stabilized by a lithiated glutathione crown (LiG‐AuNPs) on carboxyl groups, along with a demonstration of their specific ability to deliver lithium to the brain, resulting in the modulation of GSK‐3β without affecting plasma lithium levels.

The synthetic procedure is straightforward and allows for the synthesis of gold metal clusters with a very narrow size distribution of ≈2 nm, exhibiting interesting morphological, photophysical, and chemical‐biological properties. Analytical characterization of the particles determined a probable composition between the two clusters: (Li_2_‐G)_79_Au_147_ and (Li_2_‐G)_166_Au_309_, with a lithium content of 2 *wt*%. The particles exhibit a strong tendency to aggregate, even forming regular supramolecular arrays of NPs with face‐centered cubic packing, as confirmed by SAXS analysis of solid samples recovered from the synthetic procedure and by SEM analysis of samples dispersed in water, deposited on glass slides, and left to dry slowly in air. Regular polyhedral structures with a size of a few hundred micrometers have been observed under the SEM microscope as strong evidence of the regular size distribution of NPs and their tendency to aggregate. This trend has also been observed when LiG‐AuNPs are dispersed in aqueous media. Monodisperse aggregates in the size of a few hundred microns (*a*‐LiG‐AuNPs) can be obtained by simple sonication. Such aggregates are non‐toxic to several cell lines below 2 mg mL^−1^. *a‐*LiG‐AuNPs are rapidly internalized into the cell, mainly (≈65%) via clathrin‐mediated endocytosis, as already observed in TEM analysis for sodium‐bearing NPs analogs.^[^ ^3^ ^]^ After invagination into vesicles, the nanoparticles were found to be singularly dispersed throughout the cell, in the cytosol, endoplasmic reticulum, mitochondria, Golgi apparatus, and even in the nucleus. The presence of salts, proteins, and other biological molecules (e.g., glucose) could cause breakdown into single particles, an effect useful for the targeted release of lithium into the cell. When in a cation‐rich medium (e.g., a culture medium resembling that of an extracellular milieu), *a*‐LiG‐AuNPs partially release their lithium content, probably from the outer surface of the aggregate. In contrast, the fraction of lithium retained by AuNPs inside the aggregate, delivered, and released into the cell through cation exchange has been demonstrated to modulate GSK‐3β effectively. This process of delivering lithium cations into cells is more efficient than a passive diffusion process based on the ion‐transport systems. Indeed, cells do not have specific ion channels for Li^+^, and as a free ion, it enters the cells mainly via sodium channels or Na^+^/Li^+^ antiports.^[^ ^57^, ^58^ ^]^ Thus, the glutathione‐coated AuNPs device, by employing endocytosis, allows for a more rapid buildup of the lithium concentration in cells, as demonstrated by the higher intracellular lithium levels in cells treated with *a*‐LiG‐AuNPs versus LiCl. Notably, the variability of intracellular Li^+^ concentration in cells treated with *a*‐LiG‐AuNPs was smaller than that of cells treated with LiCl, thus indicating that conveying lithium through nanoparticles rather than lithium salts allows for better control of intracellular cation levels. As a result, high levels of lithium in the cell are reflected by significant increases in GSK‐3β phosphorylation at Ser9 (pGSK‐3β^Ser9^). The ability of *a*‐LiG‐AuNPs to induce GSK‐3β phosphorylation at Ser9, likely inhibiting its activity, was then extended to in vivo models. Water‐dispersed *a*‐LiG‐AuNPs were administered intranasally to adult C57Bl/6 mice to promote targeted delivery to the brain. We indeed demonstrated that, if applied intranasally, *a*‐LiG‐AuNPs may efficiently reach the brain, bypassing systemic, blood‐based administration and are able to determine an increase of pGSK‐3β^Ser9^ in situ (especially the hippocampus) in a concentration‐dependent manner, also at lithium doses (i.e., 10 mg mL^−1^) that are lower respect to those used for mood‐disorder treatment, and without affecting plasma lithium levels. Notably, intranasal administration of aggregated AuNPs could represent the best choice for drug delivery into the brain. Indeed, several studies have reported the efficacy of intranasal drug application for the brain delivery of molecules, nanoparticles, extracellular vesicles, and other substances, achieving effective concentrations.^[^ ^59^, ^60^ ^]^ Drugs are thus directly transported to the brain by axons of the olfactory nerve, thus providing a non‐invasive method of bypassing the blood‐brain barrier (BBB) to deliver therapeutic agents to the brain.^[^ ^61^, ^62^ ^]^ Moreover, it is known that olfactory bulbs project to parahippocampal brain regions (e.g., entorhinal cortex) and then to the hippocampus, thus allowing there a more precise modulation of GSK‐3β activity with respect to other brain regions (e.g., the neocortex). At the end of the administrations, lasting either 5 days or a longer period (5 months, see Methods for details), we found that total GSK‐3β levels were not significantly altered by both short‐ and long‐term intranasal *a*‐LiG‐AuNP treatment, while the pGSK‐3β^Ser9^/GSK‐3β ratio shifted in favor of the phosphorylated derivative, especially in the hippocampus in a concentration‐dependent manner, being significantly different respect to vehicles also at the lowest dose tested (10 mg/mL).

It is interesting to note that, for short‐term applications, pGSK‐3β^Ser9^ levels, evaluated ten days after the last administration of LiG‐AuNPs, remained significantly high. Our protocol for long‐term application is based on this observation. Indeed, after 5 days of intranasal administration, mice were left unadministered for 10 days, and then newly administered for a further 5 days, and so on up to 5 months. When we examined the value of the ratio pGSK‐3β^Ser9^/GSK‐3β, we did not find any differences between the values obtained at the end of the short‐ and long‐term applications. We interpreted these results as a continuous inhibition of GSK‐3β without any cumulative effects. A modest accumulation of gold was found only in the brains of mice treated for a prolonged period (5 months) at the highest administered dose (100 mg mL^−1^). Notably, the amount of gold found in the brains of the 100 mg mL^−1^‐treated mice was approximately three times higher than that found in vehicle‐treated brains; however, the amount of nanoparticles administered was more than ten times higher. Moreover, no significant accumulation was found after a 5‐month treatment with 10 mg mL^−1^. These data then suggest a possible clearance of gold from the mouse brain. Several literature reports indicate that nanoparticles, including AuNPs, are efficiently eliminated from the brain through the glymphatic system.^[^ ^49^, ^50^ ^]^ However, Zhang et al., in 2012, demonstrated that GSH‐protected AuNPs have a small size and can be efficiently metabolized by renal clearance.^[^ ^51^ ^]^ From this point of view, we found a positive correlation between the amount of gold in the brain of LiG‐AuNP‐treated mice and the amount of the same metal found in the kidneys, thus suggesting that gold is (likely) eliminated by the urinary system. However, this aspect will be further investigated in detail in the next step.

Notably, the described approach of delivering lithium to the brain through AuNPs did not induce signs of gliosis in the mouse brain, did not significantly alter plasma lithium levels, and did not affect the parameters commonly used to evaluate a mouse's wellness.^[^ ^63^ ^]^

As evidence of the modulation of GSK‐3β activity by *a*‐LiG‐AuNPs, the inhibition of the phosphorylation of the GSK‐3β’s substrate tau protein was found, also in an experimental paradigm of tau hyperphosphorylation. This aspect prompted us to evaluate a possible therapeutic application of the LiG‐AuNPs in the context of GSK‐3β‐dependent neurodegenerative illness, such as Alzheimer's disease. The data we present indicate that a 2‐month treatment with aggregates of LiG‐AuNPs is able to reverse memory impairment in the AD mouse model with advanced pathology (e.g., 12‐month‐old 3×Tg‐AD mice^[^ ^52^ ^]^). This is a significant result because it demonstrates that LiG‐AuNPs are able to rescue memory that was just affected. Even if this effect is associated with a reduced amount of total tau, and then proportionally also of pTau, lithium‐dependent mechanisms other than that involving GSK‐3β may support these beneficial effects. Indeed, it is known that Li^+^ induces synapse formation by depleting brain inositol levels through the inhibition of inositol monophosphatase (IMPase) and inositol polyphosphate 1‐phosphatase (IPPase).^[^ ^64^ ^]^

All these data suggest the potential use of this site‐specific inhibitor of GSK‐3β as a helpful tool for the treatment of brain/mental/psychiatric diseases that depend on GSK‐3β activation, such as mood disorders, or even neurodegenerative diseases such as tauopathies and Alzheimer's disease.

## 4 Conclusion

Lithium, a potent GSK‐3β inhibitor with therapeutic potential for mood disorders, neurodegenerative diseases, and viral infections, faces challenges in clinical use due to its narrow therapeutic window and toxicity at high doses. This study introduces gold nanoparticles (AuNPs) stabilized with a lithiated glutathione crown (LiG‐AuNPs) as a novel nanodevice for targeted lithium delivery to the brain. LiG‐AuNPs, synthesized with precise size control (≈2 nm) and high lithium content (2 wt%), effectively deliver lithium within cells via endocytosis, bypassing systemic circulation and overcoming blood‐brain barrier limitations through intranasal administration. In vivo studies in mice demonstrated increased GSK‐3β phosphorylation, which inhibited its activity in the hippocampus without altering plasma lithium levels or causing gliosis. The system showed promise in mitigating tau hyperphosphorylation, a hallmark of Alzheimer's disease, while ensuring safety and maintaining wellness. This nanoparticle‐based lithium delivery method offers a promising therapeutic approach for brain disorders dependent on GSK‐3β activation, including mood disorders, tauopathies, and Alzheimer's disease.

## 5 Experimental Section

### Chemicals and Consumables

Tetrachloroauric acid trihydrate (≥49.0% Au basis; Merck), reduced glutathione (GSH; 98.0%; Merck), lithium hydroxide monohydrate (LiOH; 99%; Merck), sodium hydroxide (NaOH; 98.0%; Sigma‐Aldrich), sodium borohydride (NaBH_4_; 98.0%; Merck), lithium chloride (LiCl; 99%; Merck), sodium chloride (NaCl; 99.8%; Sigma‐Aldrich), water (HPLC grade, Merck), methanol (HPLC grade, Merck), hydrogen peroxide (30%, Carlo Erba, Milan, Italy), sulfuric acid (95%, Carlo Erba), hydrochloric acid (37%, Carlo Erba), nitric acid (69.5%; Merck), gold(III) standard solution (100 mg_Au_/L in 10% hydrochloric acid; Transition metal mix 3 for ICP, TraceCERT, Merck), lithium(I) standard solution (10 mg_Li_/L in 10% nitric acid; Periodic table mix 1 for ICP, TraceCERT, Merck) were used as received without further purification procedures.

TEM grids (carbon film supported by 300‐meshes copper) were supplied by Assing (Italy). Centrifugal filters, Vivaspin 500, with a polyethersulfone membrane and a molecular weight cut‐off of 5 kDa, were provided by Merck.

### Synthesis of a‐NaG‐AuNPs and a‐LiG‐AuNPs

*a*‐NaG‐AuNPs were synthesized as described in Buonerba et al., (2020).^[^ ^3^ ^]^ *a*‐LiG‐AuNPs were synthesized with a modified procedure described in the following. A 100 mL round‐bottom flask, equipped with a magnetic stir bar, was charged sequentially at 25 °C with tetrachloroauric acid (0.333 g, 0.846 mmol), methanol (27.8 mL), water (22.2 mL), reduced glutathione (GSH, 0.581 g, 1.89 mmol), and lithium hydroxide monohydrate (0.388 g, 9.25 mmol). The addition of GSH caused turbidity, which disappeared after the addition of lithium hydroxide, ultimately yielding a clear solution. The solution was transferred into a 3 L round‐bottom flask, diluted with 260 mL of methanol and 760 mL of water, and treated rapidly with 15 mL of a freshly prepared aqueous solution of sodium borohydride (0.145 g, 3.83 mmol) under vigorous stirring at room temperature, which caused the formation of a dark brown colloidal suspension. After 48 h, lithium chloride (15.38 g, 0.363 mol) and 700 mL of methanol were added, and the colloidal suspension was transferred into a glass Imhoff cone. The *a*‐LiG‐AuNPs precipitated from the solution within 48 h. They were recovered by removing the supernatant, followed by centrifugation of the resulting suspension, which allowed for the separation of the AuNPs, which were finally dried in vacuo. Yield 0.25 g.

### Instrumental Characterization of G‐AuNPs

Wide‐angle x‐ray diffraction (WAXD) analysis was carried out in reflection mode with an automatic Bruker D8 powder diffractometer (Bruker, Billerica, MA) using the Ni‐filtered Cu‐K_α_ radiation. UV‐visible spectroscopy (UV–Vis) and fluorescence analyses were performed using a Varian Cary 50 UV–Vis spectrophotometer and a Varian Cary Eclipse spectrophotometer, respectively (Varian, Palo Alto, CA). Small‐angle x‐ray scattering (SAXS) profiles were recorded using a high‐performance SAXSess analyzer from Anton Paar KG (Graz, Austria). Data collection was performed in the slit collimation configuration with the SAXSess camera attached to a conventional x‐ray source (Cu Kα radiation, wavelength λ = 1.5418 Å). The slit‐smeared data in the SAXS region were deconvolved with the primary beam intensity distribution using the SAXSquant software to obtain the corresponding pinhole scattering (desmeared) intensity distribution. Scanning electron microscopy (SEM) images were acquired with a Phenom desktop microscope from Thermo Fisher Scientific (Thermo, Waltham, MA). The specimen was prepared as follows. An aqueous colloidal suspension of a‐LiG‐AuNPs (1 mg mL^−1^) was deposed on a glass slide, covered with a lens paper sheet, and slowly dried in air at room temperature. The sample was directly analyzed without metal sputtering. Transmission electron microscopy and scanning transmission electron microscopy (TEM/STEM) imaging of AuNPs was performed with a JEOL F200 Cold Field Emission TEM/STEM operated at 200 kV and equipped with a Rio9 Camera (Gatan Inc., Pleasanton, CA), a high‐angle annular dark field (HAADF), and an X‐ray energy dispersive spectrometer (XEDS) detector. The specimens were prepared by dispersing the samples in ethanol and depositing the suspensions onto copper TEM grids. Dynamic Light Scattering (DLS) measurements were performed using a Zetasizer Nano ZS (Malvern Instruments, Worcestershire, UK). Nanoparticles were analyzed before ultrasound sonication, as well as 1–7 days and 30 days after ultrasound sonication, in a volume of 200 µL at various concentrations. The measurements were made at a fixed position with an automatic attenuator. The temperature was maintained at 37 °C by an internal heat controller. For each condition, five measurements were averaged. Thermogravimetric analyses (TGA) were performed on a TG209F1 thermoanalyzer from Netzsch (heating rate of 10 °C min^−1^ in oxygen atmosphere). Elemental analysis was performed on a CHNS Thermo Scientific Flash EA 1112 analyzer equipped with a thermal conductivity detector.

### Cell Cultures for AuNP Treatments

The following immortalized cell lines were used in the in vitro test: hepatocellular carcinoma HepG2 cells, human neuroblastoma SH‐SY5Y cells, African green monkey kidney epithelial cells VERO, and adenocarcinoma human alveolar basal epithelial A549 cells. Specifically, HepG2 cells were cultured in Eagle's minimum essential medium (MEM) supplemented with 1% non‐essential amino acids and 2 mM l‐glutamine, as described in Buonerba et al., (2020).^[^ ^3^ ^]^ Similarly, SH‐SY5Y cells were cultured in Dulbecco's Modified Eagle Medium (DMEM): F12, VERO cells were maintained in RPMI 1640 medium (Thermo), and A549 cells were cultured in high‐glucose DMEM.^[^ ^65^ ^]^ All culture media were supplemented with 10% (v/v) fetal bovine serum (FBS) and 1% penicillin‐streptomycin‐neomycin antibiotic mixture (PSN, Thermo) and maintained at 37 °C in a humidified atmosphere of 5% CO_2_. Cells were grown in T25/T75 culture flasks, and just before use, they were plated on 35‐mm six‐well plates in their own culture media at a density of 10^6^ cells/well, suitable for WB analysis (vide infra).

### Cultures of Hippocampal Astrocytes and Neurons

Primary cultures of hippocampal astrocytes and neurons were prepared from E18 C57BL/6 mice, as previously described.^[^ ^66^ ^]^ Briefly, after brain removal, the hippocampus was dissected under a stereomicroscope in phosphate‐buffered saline (PBS) at 4 °C and then incubated for 10 min at 37 °C in a 1:1 solution of PBS and trypsin‐ethylenediaminetetraacetic acid 0.025%/0.01% w/v (Biochrom AG, Berlin, Germany). After trypsin inactivation with 1% FBS, the tissue was centrifuged and suspended in Minimum Essential Medium (MEM, Biochrom) containing 1% FBS, 1% glutamine (2 mM), 1% PSN, and glucose (25 mM). The tissue was then mechanically dissociated with a fire‐polished Pasteur pipette at room temperature (RT) and centrifuged at 235 × *g* for 10 min at RT. For astrocytes preparation, dissociated cells were suspended in DMEM supplemented with 10% FBS and 1% PSN. Astrocytes were plated on poly‐L‐lysine (0.1 mg mL^−1^) pre‐coated 35‐mm six‐well plates at a 10^6^ cells/well density for WB analysis. For neurons preparation, dissociated cells were resuspended in the previously described dissociation medium, added with 5% horse serum and 5% FBS, and plated on poly‐L‐lysine pre‐coated 20‐mm coverslips (10^5^ cells/well) for immunocytochemistry. After 24 h from seeding (1 day in vitro, DIV), the culture medium was replaced with a fresh medium consisting of Neurobasal medium (Thermo), 2% B‐27 (Thermo), 2 mM glutamine and 1% PSN. At 4 DIV, this medium was replaced with a glutamine‐free version of the same medium, and the cells were grown for 10 more days before carrying out experiments. All cultured experimental models were treated with either *a*‐NaG‐AuNPs or *a*‐LiG‐AuNPs at various concentrations and for multiple times (as described below) in their culture medium.

### Animals and Experimental Groups

C57BL/6 and 3×Tg‐AD mice were used for in vivo experiments. All mice were maintained in the animal house at Università Cattolica del Sacro Cuore (Rome, Italy) under a 12‐h light‐dark cycle at RT without limited food and water. C57BL/6 male mice of 3 months of age were used and treated for 5 days up to 5 months. Mice were maintained in the animal house at Università Cattolica del Sacro Cuore (Rome, Italy) under a 12‐h light‐dark cycle at RT without limited food and water. Mice were divided into the main experimental groups and kept in separate cages according to *a*‐LiG‐AuNPs treatment concentrations (0‐vehicle; 1, 10, and 100 mg/mL; n = 5–10 animals/group). Mice were treated for 5 consecutive days with either vehicle or *a*‐LiG‐AuNPs dissolved in water at a concentration of 1, 10, and 100 mg/mL, administered intranasally (3 µL/nostril/day; see Figure S1, Supporting Information). Intranasal administration was performed according to the procedures described by Hanson L. R. et al., (2013) with minor modifications.^[^ ^67^ ^]^ Mice were sacrificed by cervical dislocation on the last day of treatment, 6 h after the last dose of *a*‐LiG‐AuNPs, except for some of those treated with 100 mg mL^−1^ LiG‐AuNPs by intranasal administration, which were sacrificed 10 days after the last treatment dose. A subset of mice (n = 4 for each experimental group) was subjected to a “long‐term” treatment, consisting of intranasal application of a‐LiG‐AuNPs or vehicle twice a month (5 consecutive days every 2 weeks, see Supplementary Figure 1) for 5 months before sacrifice. After sacrifice, all mice were decapitated, and their brain was removed: one hemisphere underwent lyophilization to determine gold and lithium content in the brain by inductively coupled plasma‐optical emission spectroscopy (ICP‐OES see below), while hippocampus, neocortex, and olfactory bulb of the contralateral hemisphere were explanted for WB analyses (see below). Before sacrifice, blood (200–300 µL) was collected via retro‐orbital withdrawal to determine lithium plasma concentration.

3×Tg‐AD mice were used for in vivo therapeutic application of *a*‐LiG‐AuNPs.^[^ ^52^ ^]^ Female mice of 12 months of age were used and treated for 2 months, according to the previously described experimental paradigm for long‐term administration (see Figure S2, Supporting Information), with vehicle or *a*‐LiG‐AuNPs 100 mg/mL (n = 4‐5 animals/group). As previously described, at the end of treatment, mice were subjected to behavioral tests and then blood and brain tissues were collected for WB and ICP‐OES analyses.

### Cell viability

Cell viability was assessed in SH‐SY5Y and VERO cells using both the trypan blue exclusion method and the MTT assay. Trypan blue is a dye (Sigma‐Aldrich; used at 0.4%) able to cross the cell membrane of dead cells, which becomes blue. Cell counts have been carried out using a Burker chamber. Cell viability has been estimated as the percentage of living cells to dead cells (blue) × 100. For the MTT (3‐[4,5‐dimethyltiazol‐2‐il]‐2,5‐diphenyl tetrazolium bromide, Sigma‐Aldrich) assay, cells were plated onto a 96‐well plate (1.5×10^4^ cells/well) for 24 h. The next day, the cells were treated with *a*‐LiG‐AuNPs at increasing concentrations in 100 µL of culture medium without phenol red for an additional 24 h. After NPs treatment, 10 µL MTT solution (5 mg mL^−1^ in phenol red‐free RPMI‐1640 medium) were added to the previous solution, and the cells were incubated for a further 2 h. After this, 100 µL of MTT solvent (HCl 0.1 N in anhydrous isopropanol, Sigma‐Aldrich, Burlington, MA) and the formed formazan crystals were dissolved by gentle trituration. Spectrophotometric analysis was carried out by measuring the absorbance at 570 nm after subtracting the absorbance at 690 nm.

### Western Blot Analyses on Cells and Brain Tissues

Cells were treated with either vehicle or *a*‐LiG‐AuNPs (1 and 0.05 mg mL^−1^) or LiCl (6, 3, and 0.15 mM) for 1 or 24 h before being collected. In some cases, described in the text, *a*‐NaG‐AuNPs were used. Hippocampi, cortices, and olfactory bulbs were explained from C57BL/6 mice treated with either vehicle or *a*‐LiG‐AuNPs (1, 10, and 100 mg mL^−1^). Cultured cells and tissues from animals were homogenized in RIPA buffer, supplemented with 1 mM phenylmethylsulphonyl fluoride (PMSF), sodium fluoride, sodium orthovanadate, and protease inhibitor (PI) mixture. Then, they were sonicated and centrifuged at 13000 × *g* for 20 min at 4 °C. Bradford protein assay was used to determine the protein concentration of the collected supernatants. For each sample, an equivalent amount of protein (30 µg) was loaded onto 8% tris‐glycine polyacrylamide gel for electrophoresis separation. Proteins were electroblotted onto nitrocellulose membranes and then blocked with 5% non‐fat dry milk in tris‐buffered saline containing 0.1% Tween‐20 for 1 h at RT. Membranes were incubated with the following primary antibodies: rabbit anti‐phospho‐GSK‐3β (Ser9) (#9336, Cell Signaling, Danvers, MA, USA), rabbit anti‐GSK‐3β (#12 456, Cell Signaling), mouse anti‐GFAP (#3670, Cell Signaling), mouse anti‐pTau^T205^ (# MN1020, Invitrogen), mouse anti‐pTau^Ser396^ (#35‐5300, Invitrogen), mouse Tau13 (sc‐21796, Santa Cruz Biotechnology, Dallas, TX, USA), all used at dilution 1:1000, overnight at 4 °C. Mouse anti‐GAPDH (#ab8245, Abcam; 1:1000), and Ponceau S Staining Solution (Thermo) were used as loading controls. Membranes were then incubated with appropriate secondary horseradish peroxidase‐conjugated (HRP) antibodies diluted at 1:5000 (anti‐rabbit #7074, anti‐mouse #7076; Cell Signalling) for 1 h at RT. Visualization was performed with WESTAR ECL ETA C ULTRA 2.0 (XLS075, Cyanagen, Bologna, Italy), using UVItec Cambridge Alliance. Molecular weights for immunoblot analysis were determined through Precision Plus Protein Standards (BioRad, Hercules, CA, USA). Densitometric analysis was carried out with UVItec software. Experiments were repeated at least three times.

### Immunofluorescence (IF) and Differential Interference Contrast (DIC) Microscopy on Cultured Cells

After vehicle or *a*‐LiG‐AuNPs treatments, cells were fixed in formalin solution (10% in PBS) for 15 min. For IF, non‐specific binding sites were blocked by 20 min incubation of cells with bovine serum albumin (BSA, 0.3% in PBS). After this blockade, cells were incubated overnight with a specific anti‐tau antibody (mouse anti pTau^Thr205^) or the combination of the antibodies anti‐MAP2 (#M9942, Sigma) and anti‐Synapsin‐1 (#5297, Cell Signaling), and then labelled with the specific secondary antibodies (goat anti‐mouse Alexa Fluor 488, and donkey anti‐ mouse Alexa Fluor 546 and donkey anti‐rabbit Alexa Fluor 488) for 90 min at RT. Cell nuclei were labelled by 15‐min incubation with 4,6‐diamidine‐2‐phenylindole dihydrochloride (DAPI, 0.5 µL mL^−1^). DIC microscopy images (512 × 512 pixels at 63× magnification) were obtained in transmission mode using a Leica TCS‐SP5 laser scanning system, which operated with laser light at a wavelength of 546 nm. IF images were collected with a Nikon A1MP confocal system at 60× magnification (1024 × 1024 pixels).

### Determination of Gold and Lithium Concentration by ICP‐OES

The concentrations of gold and lithium in solid samples, culture media, cultured cells, tissues, and brains were determined using inductively coupled plasma‐optical emission spectroscopy (ICP‐OES) with a Thermo iCap 6000 analyzer from Thermo. Instrumental calibration was performed using standard solutions of gold(III) (100 mg_Au_/L in 10% hydrochloric acid) and lithium(I) (10 mg_Li_/L in 10% nitric acid). AuNP samples were mineralized before being analyzed by ICP‐OES to determine gold and lithium content as follows. 2.5 mL of concentrated sulfuric acid (95 wt%) were added to 10 mg of the sample in a Kjeldahl flask, and the resulting suspension was heated to 250 °C for 30 min; 4.0 mL of hydrogen peroxide (30 wt%) were thus added at RT, and the resulting suspension was heated to 250 °C to produce a clear solution containing metallic gold nuggets. 1.5 mL of aqua regia were added at RT for dissolving the gold nuggets, and the resulting clear solution was diluted with hydrochloric acid (10 vol%) to a final volume of 10 mL.

The amount of lithium released from the LiG‐AuNPs in MEM was determined as follows: 30 mg of LiG‐AuNPs were suspended in 30 mL of MEM and sonicated for 10 min. Aliquots of the suspension were sampled after 1 and 24 h, centrifuged at 5000 rpm for 5 min using centrifugal filters with a molecular weight cut‐off of 5 kDa, and the filtrate was analyzed by ICP‐OES. The uptake of lithium in human neuroblastoma SH‐SY5Y was determined by ICP‐OES analysis of acidic digested cells incubated for 24 h with 1 mg mL^−1^ of *a*‐LiG‐AuNPs (3 mEq L^−1^ lithium) and 6 mM LiCl (6 mEq L^−1^ lithium). A Kjeldahl flask was charged with the cell pellet redispersed in deionized water, 1.0 mL of concentrated sulfuric acid (95 wt%), and warmed up to 250 °C for 30 min. After cooling to RT, 1 mL of hydrogen peroxide (30 wt%) was slowly added to the flask and then warmed to 250 °C until a clear solution was obtained. Finally, the sample was diluted with aqueous nitric acid (10%) and analyzed by ICP‐OES.

Mice brains were mineralized before being analyzed by ICP‐OES to determine gold and lithium content as follows. 2.0 mL of concentrated sulfuric acid (95 wt%) were added to the previously lyophilized and weighed brain sample in a Kjeldahl flask and treated at 250 °C for 30 min. 4.0 mL of hydrogen peroxide (30 wt%) were then added at RT, and the resulting suspension was heated to 250 °C to produce a clear solution. 1.5 mL of aqua regia were added at RT to dissolve the metallic gold, and the resulting clear solution was diluted with hydrochloric acid (10 vol%) to a final volume of 5 mL and analyzed by ICP‐OES.

### Determination of Lithium Concentration in Plasma

Lithium levels in the plasma of *a*‐LiG‐AuNP‐treated animal were measured by Atellica CH Lithium (Li) assay for in vitro diagnostic use in the lithium quantitative determination; the method is a colorimetric endpoint applied on Atellica CH Analyzer. The CV% intra‐ and inter‐assay at 0.60 mmol L^−1^ were 2.2% and 4.0%, respectively.

### TEM Analyses of Cells

Specimens for TEM imaging of Hep G2 cells incubated with AuNPs were prepared as described below. After incubation of the Hep G2 cells with AuNPs (700 and 70 µg mL^−1^ in a final volume of 1 mL), the cells were fixed for 30 min at RT in 2% glutaraldehyde in cacodylate buffer (0.1 mol L^−1^, pH 7.4), then scraped and pelleted. The pellets were additionally fixed for 24 h at 4 °C and then treated for 1 h with a staining solution prepared by combining equal volumes of an aqueous osmium tetroxide solution (4%) in cacodylate buffer (0.3 m) and an aqueous potassium ferricyanide solution (3%). The cell pellets were washed with deionized water at RT and treated with aqueous osmium tetroxide (2 wt%) for 30 min. After several washings with bi‐distilled water, the pellets were treated overnight at 4 °C with an aqueous uranyl acetate solution (1%). The samples were dehydrated by sequential treatment with ethanol solutions with increasing alcohol concentration (70%, 80%, 90%, and 100%) and with neat acetone for 10 min each, and finally embedded in Epon epoxy resin and cured at 60 °C for 48 h. Thin sections of the resin‐embedded cells were obtained using a UC7 ultramicrotome from Leica, collected onto copper grids, and analyzed with a Tecnai Spirit transmission electron microscope from FEI, operating at an acceleration voltage of 120 kV and equipped with a LaB_6_ thermionic source and a twin objective lens. Images were acquired using a Gatan Orius CCD camera (Pleasanton, CA).

### Behavioral Tests

Novel Object Recognition (NOR) and Y‐maze paradigms were performed. In the NOR test, on day one, animals were familiarized for 10 min to the test arena (45 × 45 cm). On day 2 (training session), two identical objects were placed symmetrically in the central part of the arena, and each mouse was allowed to explore for 10 min. On day 3 (test session), a new object replaced one of the old objects. The animals were allowed to explore for 5 min, and a preference index (PI), calculated as the ratio between the time they spent exploring the novel object and the time spent exploring both objects, was used to measure recognition memory. For the evaluation of spatial working memory, we employed the Y‐maze test. The animals were allowed to explore the 3 arms of a Y‐shaped apparatus for 8 min, made of non‐transparent white plexiglas. The number and order of entrances within the 3 different arms were recorded. Spontaneous alternation behavior was then quantified as the percentage of the number of correct triplets, defined as sequential entrances within the 3 different arms, divided by the total number of entrances.

### Determination of Animal Wellness

In accordance with the Italian Ministry of Health guidelines (Legislative Decree No. 116/1992 and 26/2014) and European Union (Directive No. 86/609/EEC) legislation on animal research (see below), animal wellness was evaluated during LiG‐AuNP treatment. Among the parameters studied, we specifically monitored weight loss, fur condition, excessive grooming, anxiety, and cognitive conditions.

### Statistical Analysis

Statistical comparisons and analyses were performed using SigmaPlot software version 14.0. Data samples were subjected to a normal distribution assay and then expressed as mean ± standard error of the mean (SEM). The Mann‐Whitney (Wilcoxon) nonparametric statistic was used when experimental data were fewer than 10 observations (e.g., densitometric analysis of WB data). The level of significance (p) was set at 0.05. Statistical analyses used are described in the figure legends.

### Ethics statement

The Ethics Committee of Università Cattolica approved all animal procedures, which were fully compliant with Italian (Ministry of Health guidelines, Legislative Decree No. 116/1992 and 26/2014) and European Union (Directive No. 86/609/EEC) legislation on animal research. Authorization No. 594/2022‐PR from the Italian Ministry of Health. Animals were supplied by the Division of Animal Resources of Università Cattolica.

## Conflict of Interest

The authors Roberto Piacentini, Antonio Buonerba, Alfonso Grassi, and Claudio Grassi declare a competing interest given that they patented the LiG‐AuNPs with international application number PCT/IB2024/050915 and international publication number WO 2024/165949 A1.

## Supporting information



Supporting Information

## Data Availability

The data that support the findings of this study are available from the corresponding author upon reasonable request.
