# Supplementary material for: Lithium‐Charged Gold Nanoparticles: A New Powerful Tool for Lithium Delivery and Modulation of Glycogen Synthase Kinase 3 Activity
Source: Adv Mater. 2025 Sep 29;38(3):e13858. doi: 10.1002/adma.202513858 (PMC12801361; doi:10.1002/adma.202513858)
Supplement: Supplementary file 1 — Supporting Information [file ADMA-38-e13858-s001.docx]

Supporting Information

Lithium-Charged Gold Nanoparticles: A New Powerful Tool for Lithium Delivery and Modulation of Glycogen Synthase Kinase 3 Activity

Antonio Buonerba^†^*, Giulia Puliatti^†^, Domenica Donatella Li Puma, Bruno Bandiera, Beatrice Cannata, Maria Elena Marcocci, Nicolina Castagno, Irene Contento, Salvatore Impemba, Mariarosa Scognamiglio, Rocco Di Girolamo, Vincenzo Naddeo, Patrizia Canton, Carmine Capacchione, Laura Sposito, Martina Albini, Francesco Pastore, Silvia Baroni, Alfonso Grassi, Claudio Grassi and Roberto Piacentini*

^a^ Department of Chemistry and Biology “Adolfo Zambelli”, University of Salerno, Via Giovanni Paolo II, 84084 Fisciano (SA), Italy.

^b^ Department of Neuroscience, Università Cattolica del Sacro Cuore, 00168 Rome, Italy.

^c^ Fondazione Policlinico Universitario A. Gemelli, IRCCS, Rome, Italy.

^d^ Department of Public Health and Infectious Diseases. Sapienza University of Rome, 00185 Rome, Italy.

^e^ Department of Chemistry, University of Naples Federico II, Naples, Italy.

^f^ Department of Civil Engineering, University of Salerno, Via Giovanni Paolo II, 84084 Fisciano (SA), Italy.

^g^ Department of Molecular Sciences and Nanosystems, University Ca’ Foscari Venezia, Dorsoduro 3246, 30123 Venezia, Italy

^h^ Department of Basic biotechnological sciences, intensivological and perioperative clinics, Università Cattolica del Sacro Cuore, Largo F. Vito 1, 00168, Rome, Italy

^†^ These authors equally contributed to the work.

* Corresponding authors: A. Buonerba ([abuonerba@unisa.it](mailto:abuonerba@unisa.it)) and R. Piacentini ([roberto.piacentini@unicatt.it](mailto:roberto.piacentini@unicatt.it))


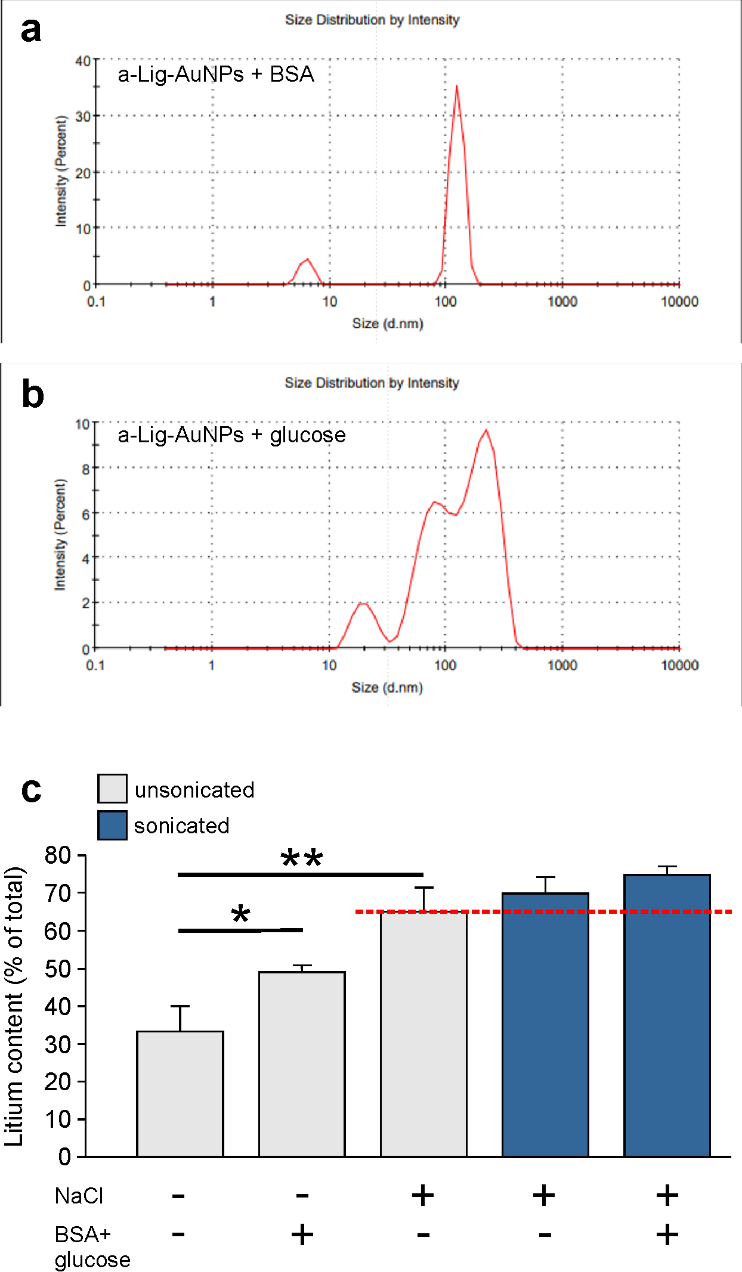


***Figure S1: Aggregates of LiG-AuNPs preserved internal nanoparticles from releasing lithium in the medium. (a, b)*** *DLS analysis of a-LiG-AuNPs treated with (a) BSA and (b) glucose, showing the rupture of large aggregates and the formation of smaller aggregates (<200 nm diameter).* ***(c)*** *Bar graph showing the amount of lithium released by a-LiG-AuNPs in aqueous solution added (+) or not (–) with NaCl or BSA, and glucose. Statistical significance was assessed by ANOVA on ranks.*

**a**

**b**

**Figure S2. Experimental paradigms of LiG-AuNP treatment of living mice.** **(a)** Short-term treatment: mice were treated for 5 consecutive days with either vehicle or a-LiG-AuNPs administered intranasally (3 µL/nostril/day). Mice were sacrificed on the last day of treatment, 6 h after the last dose of a-LiG-AuNPs, and blood and brain were collected. In a subset of experiments, some mice treated with 100 mg/mL LiG-AuNPs by intranasal administration were sacrificed 10 days after the last treatment dose to collect their brains. **(b)** Long-term treatment: mice were treated for 5 consecutive days with either vehicle or a-LiG-AuNPs, intranasally administered (3 µL/nostril/day). The 5-days administration was repeated every 9 days (for a total of two 5-days administration/month) up to 5 months. Then, at the end of the fifth month mice received the final 5-days administration and then they were sacrificed 6 h after the last dose of a-LiG-AuNPs in order to collect their blood and brains.


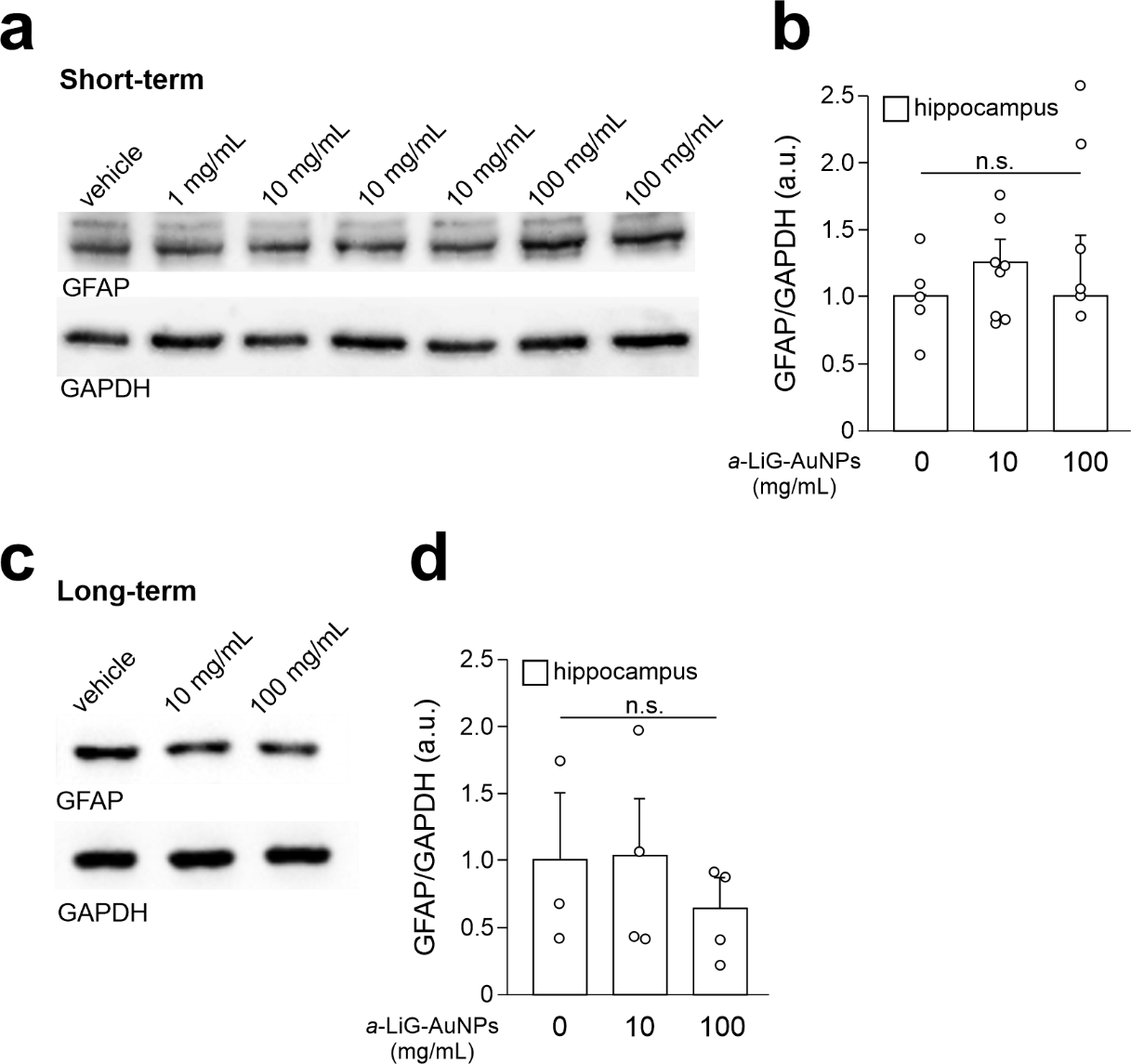


**Figure S3. Intranasal administration of a-LiG-AuNPs did not induce gliosis in the hippocampus of treated mice, neither in the short term nor in the long term. (a, c)** Western Blot analyses of hippocampal tissue of mice treated intranasally with a-LiG-AuNPs at various concentrations for 5 days (short-term) and 5 months (as described in Supplementary Figure S2; Long-term) and probed with an antibody anti-GFAP. GAPDH was used as a loading control. **(b, d)** Bar graph quantifying the WB analysis represented in panels (a, c). n.s. means not significant difference. Statistical significance was assessed by ANOVA on ranks.


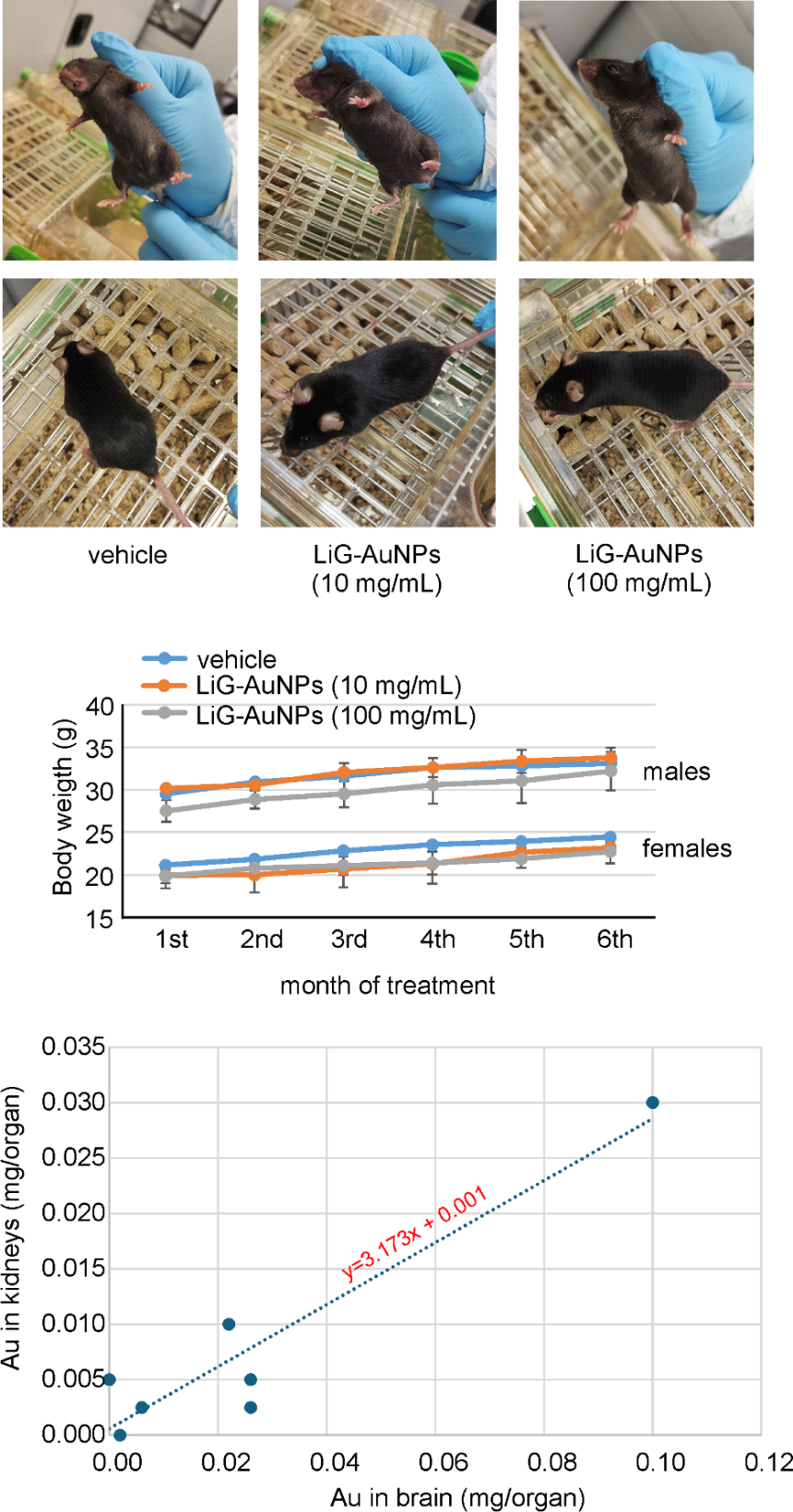


**a**

**b**

**Figure S4. Long-term intranasal administration of a-LiG-AuNPs did not induce any signs of sickness in treated mice. (a)** Representative picture of treated mice showing the normal state of the coat. **(b)** Graph showing the trend of body weight of mice (both males and females) subjected to intranasal administration of a-LiG-AuNPs for a long time (as described in Supplementary Figure S2). No significant differences were found in mice of the same sex among the three different experimental groups. **(c)** Linear regression graph showing the correlation between the amount of gold found in the brain and kidneys of a-LiG-AuNPs-treated mice.


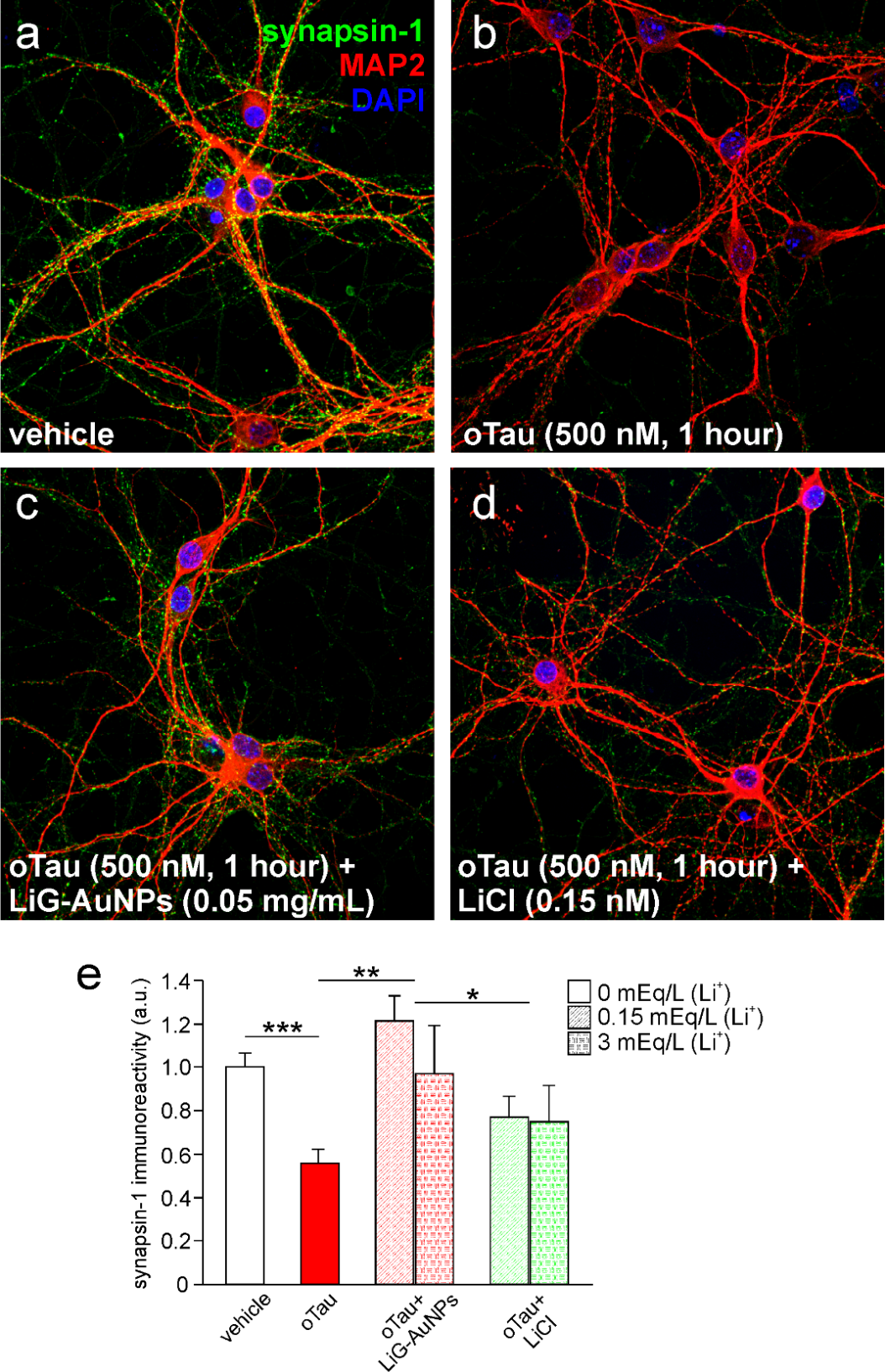


***Figure S5. a-LiG-AuNPs treatment reduced tau-induced synaptic impairment in cultured neurons. (a-d)*** *Representative images of cultured neurons stained for Synapsin-1 and MAP2, treated for 1 hour with vehicle (a), 500 nM tau oligomers (oTau, b), 500 nM oTau and 0.05 mg/mL a-LiG-AuNPs (c), or 500 nM oTau and 0.15 mM LiCl (d). DAPI was used to counterstain nuclei.* ***(e)*** *Bar graph quantifying the IF analysis represented in panels (a-d), along with the following experimental conditions: 500 nM oTau and 1 mg/mL a-LiG-AuNPs or 500 nM oTau and 3 mM LiCl. Statistical significance was assessed by ANOVA on ranks.*

**SAXS Analysis**

The (111) and (220) SAXS correlation peaks position observed in the scattering profile were compatible with a face-centered-cubic (*fcc*) where *a*=*b*=*c*, α = β = γ = 90°.

The following relation between interplanar spacing and Miller indices can be used for cubic systems:

$d_{hkl}=\frac{a}{\sqrt[2]{h^{2}+k^{2}+l^{2}}}$ (eq.1)

In the case of the sample NaG-AuNPs, from the position (*q*_1_=2.95 nm^-1^) of the first correlation peak with Miller indices *hkl* (111), the interplanar spacing can be calculated as follows:

$$d_{111}=2п/q_{1}$$

*d*_111_ *=* 2,13 nm

The *a-axis* has been determined from Eq. 1 as follows:

*a*=$d_{111}\sqrt[2]{3}$ =$2,13\sqrt[2]{3}$= 3,69 nm

For the LiG-AuNPs sample:

*a*=3,96 nm
